# The functional neurobiology of dispositions towards negative emotions

**DOI:** 10.1038/s41467-026-74565-0

**Published:** 2026-06-27

**Authors:** M. Sicorello, P. J. Gianaros, A.G.C Wright, B. Petre, T. E. Kraynak, S. B. Manuck, C. Schmahl, T. D. Wager

**Affiliations:** 1 Department of Psychosomatic Medicine and Psychotherapy, Central Institute of Mental Health, Medical Faculty Mannheim, Heidelberg University, Heidelberg, Germany; 2 German Center for Mental Health (DZPG), Partner Site Mannheim-Heidelberg-Ulm, Heidelberg, Germany; 3 Department of Psychology, University of Pittsburgh, Pittsburgh, PA USA; 4 Department of Psychology, University of Michigan, Ann Arbor, MI USA; 5 Department of Psychiatry, University of Michigan, Ann Arbor, MI USA; 6 Eisenberg Family Depression Center, University of Michigan, Ann Arbor, MI USA; 7 Department of Psychological and Brain Sciences, Dartmouth College, Hanover, NH USA; 8 Department of Psychiatry, University of Pittsburgh, Pittsburgh, PA USA

**Keywords:** Personality, Neural decoding, Diagnostic markers, Emotion

## Abstract

People differ in their tendency to experience negative emotions. This variability is largely captured by broad psychological constructs like neuroticism, whose facets include anxiety, depression, and stress vulnerability, among others. The amygdala and salience network have been assumed to underlie such dispositions, despite inconsistent evidence. We preregistered a comprehensive test of these and other competing hypotheses—accompanied by theory-agnostic machine learning prediction—using neural responses in the two most common emotional neuroimaging tasks (scenes and faces; *N* = 338/424). Evidence including Bayes factors indicated that neuroticism is not associated with any region, network, affective signature, or machine learning pattern, including the amygdala. Still, a brain-wide machine learning pattern robustly predicted the neuroticism facet *stress vulnerability* (r = .21), replicated in an independent dataset (r = .19). Predictive performance most strongly depended on somatomotor and visual networks, rather than salience network regions, relating stress vulnerability to cortical perception–action systems. Together with a multiverse analysis spanning 14 trait constructs and 1,176 models, our findings demonstrate the highly selective predictability of emotional dispositions from brain responses to common affective tasks. Therein, they highlight the importance of construct and task selection, while challenging the role of the most commonly used neural markers, including responses of the amygdala and salience network.

## Abstract (web summary)

This study refines how emotional dispositions are mapped in the brain, showing selective rather than general predictability from common fMRI tasks and challenging standard neuro-affective markers, including the amygdala and salience network.

## Introduction

People differ in their tendency to experience negative emotions, which is reflected in all contemporary models of human personality and psychopathology under various labels like “negative affectivity”, “neuroticism”, “negative affect”, and “affective instability”^1–6^. As a cornerstone of dominant dimensional taxonomies for mental disorders^7–9^, identifying the biological basis of negative affectivity is crucial for refining theoretical models by providing physical counterparts to otherwise unobservable latent trait constructs^10^. This serves the discovery of novel targets for personalized biologically oriented interventions such as psychotropic medication, neurofeedback and (non-)invasive brain stimulation^2^. Still, despite extensive theoretical contributions and empirical research, the neurobiology underlying negative affectivity remains largely unclear.

**Fig. 1:**
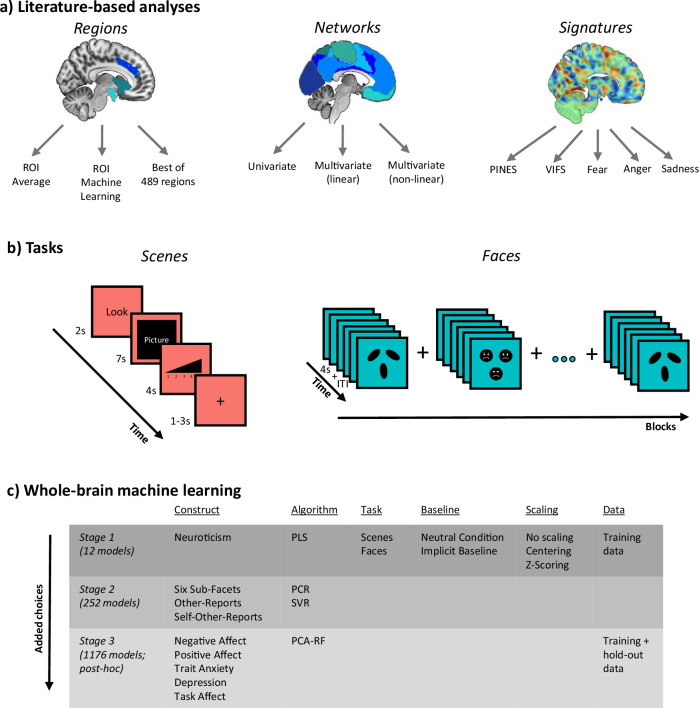
Study design and analytic workflow. Schematic illustration of the study design. **a** Literature-based analyses targeting task-based fMRI responses of anatomical brain regions, canonical resting-state networks, and affective neural signatures. The three ROIs (amygdala, anterior insula, dACC) are extracted from different sagittal slices and projected on the same slice for visual simplicity. ROI = region of interest, PINES = Picture Induced Negative Emotion Signature, VIFS = Visually Induced Fear Signature. **b** Structure of scenes and faces task. Schematic stimuli are shown for illustrative purposes to avoid copyright restrictions associated with the original image stimuli. **c** Whole-brain machine learning models across three sequential analytic stages. The first two stages were preregistered. Each stage adds modeling choices to the previous stages (e.g., additional constructs, algorithms, or using the full data). The additional constructs of stage 2 are strongly linked to neuroticism. “Six Sub-Facets” includes the facets of neuroticism: depression, anxiety, stress vulnerability, anger, impulsiveness, and self-consciousness. “Other-reports” refer to neuroticism scores provided by informants, while “Self-Other-Reports” combine these with traditional self-reports (see methods section for details). PLS = Partial Least Squares Regression, PCR = Principal Component Regression, SVR = Support Vector Regression, PCA-RF = Principal Component Analysis with Random Forest Regression.

There are three common levels of neural targets for negative affectivity, which differ in complexity and spatial scale: (1) Single brain regions^3,5^, (2) large-scale networks based on resting-state data^11^, and (3) validated whole-brain neural signatures (Fig. 1a). Theoretical and empirical work has mostly focused on salience-related brain regions and networks as a potential neural basis of negative affectivity, with emphasis on the amygdala, anterior insula, dorsal anterior cingulate cortex (dACC), and the salience network (neural levels 1 and 2; Fig. 1a)^5,12–14^.

Most of these neural targets were motivated by process-oriented studies on negative affective *states* (i.e., brief momentary experiences), which estimate “within-person” effects by comparing the same individuals across conditions (e.g., negative vs neutral stimuli). This literature is often taken to imply that neural systems supporting momentary negative affective states are also core substrates of negative affective *traits*, that is, “between-person” differences in relatively stable dispositions. However, cross-level inference from states to traits is not guaranteed and can be misleading^15,16^. Moreover, even at the state level, these salience-related regions and networks show only moderate sensitivity and limited specificity for negative affective experiences^11,17–27^. Together, these considerations motivate direct tests of brain–trait associations, including both commonly used neural markers of negative affective states as well as novel targets.

Concerning negative affectivity traits, several meta-analyses reported group differences between people with affect-related mental disorders and healthy controls for neural salience regions based on differences in structural measures, resting-state functional connectivity, and task-evoked responses to negative stimuli^28–30^. Still, more direct meta-analyses on clearly delineated dimensional negative affectivity traits do not confirm these findings^31–36^. On the one hand, large-scale studies raised doubts concerning the general predictability of individual differences like negative affectivity based on structural and resting state functional neuroimaging data^37,38^. On the other hand, psychological theory and evidence from momentary assessments in daily life indicate that negative affectivity largely manifests in an increased reactivity to stressful or threatening environments^5,39–41^. Hence, it is likely that neural correlates of negative affectivity can mainly be observed in appropriate task contexts, psychologically aligned with the specific construct of interest^1^.

Besides leveraging experimental tasks, a neurobiological understanding of negative affectivity might be improved by using state-of-the-art neural markers of momentary affective states. Affective neural signatures are innovative multi-system machine learning-based brain patterns which effectively predict specific self-reported affective experiences during experimental tasks, robustly generalize to new samples, have good test-retest reliability, and typically include regions across multiple canonical networks (brain level 3, Fig. 1a)^17,21,23,25,42,43^. Despite these advantages over single regions and networks, making neural signatures the best current targets for functional brain-based biomarkers^44^, affective neural signatures have rarely been applied to explain individual differences in affective functioning. Only one mega-analytic study showed no group differences in a clinical case-control design for negative affectivity^45^. Nevertheless, a dimensional approach covering the full spectrum of negative affectivity traits is still missing. This entails not only a large continuous range of trait levels, from very low to very high, but also a range of different trait constructs, which are often used interchangeably despite containing both shared and specific aspects of affective functioning^46^ (e.g., anxiety, neuroticism, depression).

The goal of this preregistered study was to comprehensively test how well negative affectivity is reflected by the three outlined neuro-organizational levels—single regions, canonical resting-state networks, and validated neural signatures (Fig. 1a)—and compare them to data-driven machine learning models drawing on brain-wide distributed signals. All neural measures were based on responses to negative stimuli in the most widely used affective fMRI task: viewing pictures of negative faces and scenes (Fig. 1b). This mirrors the theoretical assumption that negative affectivity results from generally heightened neural responsivity to negative stimuli and events across contexts^3,12,14^ and enhances the generalizability to the majority of the task-based fMRI literature on negative affectivity. These analyses were performed on a large community sample (*N* = 338/424; Table 1) with representative variance on neuroticism based on U.S. population norms^47^ (for descriptive statistics and correlations between all negative affectivity measures, see Table 2).

**Table 1:** Sample characteristics

	Faces (*N* = 424)	Scenes (*N* = 338)
Women (%)	52.1	48.8
Age (years)	42.8 ± 7.4	41.3 ± 7.1
Race (%)		
White	83.5	77.7
African-American/Black	15.1	19.0
Multi-racial	1.4	1.2
School years completed	17 ± 2.8	16.6 ± 3

± indicates the standard deviation. Participant sex was based on self-reports.

**Table 2:** Descriptive statistics for measures of negative affectivity

Construct	*M*	*SD*	1	2	3	4	5	6	7	8	9	10	11	12	13
1. Neuroticism	75.5	21.7													
2. N1: Anxiety	13.7	5.2	0.81												
3. N2: Hostility	12.0	5.0	0.74	0.52											
4. N3: Depression	11.5	5.6	0.84	0.63	0.54										
5. N4: Self-Consciousness	13.8	4.5	0.71	0.55	0.36	0.55									
6. N5: Impulsiveness	15.4	4.6	0.59	0.34	0.39	0.35	0.25								
7. N6: Stress Vulnerability	9.1	4	0.80	0.6	0.51	0.67	0.52	0.37							
8. N: Other-Report	16.2	9.1	0.42	0.36	0.37	0.36	0.27	0.19	0.34						
9. Negative Affect	15.8	5.3	0.62	0.53	0.45	0.62	0.38	0.30	0.48	0.29					
10. STAI	32.5	8.4	0.71	0.58	0.49	0.71	0.47	0.30	0.63	0.39	0.59				
11. BDI	4.2	4.4	0.44	0.31	0.32	0.50	0.26	0.23	0.36	0.33	0.45	0.62			
12. Positive Affect	34.5	6.5	−0.33	−0.21	−0.22	−0.32	−0.28	−0.12	−0.37	−0.13	−0.11	−0.37	−0.29		
13. Extraversion	115	19.5	−0.33	−0.23	−0.18	−0.31	−0.39	−0.04	−0.36	−0.11	−0.21	−0.30	−0.17	0.49	
14. Task-Ratings	2.4	0.7	0.03	0.10	−0.03	−0.01	0.12	−0.07	0.03	−0.02	0.02	−0.03	−0.10	−0.02	0.09

Task-Ratings = Person-wise average difference for affective task ratings between negative and neutral scenes.

*STAI* State-Trait Anxiety Inventory (trait scores), *BDI* Beck Depression Inventory.

First, we focused on literature-based associations between neuroticism and single brain regions, canonical resting-state networks, and validated affective neural signatures, testing widespread ideas from the current and past literature (Fig. 1a). We started with neuroticism as a well-studied exemplar of negative affectivity as the main outcome due to its strong theoretical basis, its large associations with other negative affectivity constructs, its relevance for mental and physical health, and its hierarchical sub-facets which allow more fine-grained approaches^4,13,48,49^.

Next, we performed preregistered data-driven machine learning analyses based on whole-brain data to derive a multivariate signature for negative affectivity and validate it in a hold-out sample (Fig. 1c, stage 1). Traits like neuroticism are higher-order constructs, which possibly do not match the abstraction level of specific task-based neural measures^1^. Therefore, we preregistered to first predict neuroticism and, if an accuracy threshold is not surpassed, test the predictability of the six lower order facets of neuroticism before evaluating the best model in the hold-out sample (depression, anxiety, stress vulnerability, anger, impulsiveness, self-consciousness; Fig. 1c, stage 2). This best model was further assessed for its psychometric properties (reliability and validity), psychological and neurobiological interpretability, and its relation to literature-based neural measures. These steps are crucial to provide a viable neural marker.

Lastly, we present extensive exploratory multiverse analyses^50^ on the predictive accuracy of ≈1200 models across a larger set of 14 affective trait constructs as well as other design and data analytic choices. The results are available via an interactive online app to allow the free exploration of factors influencing the predictability of negative affectivity traits from brain data (Fig. 1c, stage 3).

In the preregistration, we hypothesized that a whole-brain predictive pattern for neuroticism would outperform region-, network-, and signature-based models. Given the proposed importance of aligning fMRI tasks with psychological constructs, we expected this pattern to (1) show greater accuracy for the scenes task than the faces task, as the latter might be targeting emotion recognition more closely than emotional experiences; (2) correlate more strongly with negative affect than with positive affect or extraversion (construct validity); and (3) primarily reflect withdrawal-related sub-facets of neuroticism, including depression, anxiety, and stress vulnerability, as these might be more strongly reflected by the employed tasks.

## Results

### Evidence against meaningful neuroticism associations with literature-based neural measures

We first tested the association between neuroticism and neural responses across regions, networks, and validated neural signatures (Fig. 1a). The region approaches comprised average responses and local machine learning patterns in three theory-derived regions (amygdala, anterior insula, and dACC). It also included a brain-wide “best region” approach, where the region most highly correlated to neuroticism is identified in one half of the data and then tested in the other half. The network approaches comprised average responses in seven major canonical networks^51^ as well as linear and non-linear combinations of network responses, based on neural network theories of emotion^52^. Lastly, we included five well-validated neural signatures targeting negative affect (PINES)^21^, fear (VIFS)^23^, and three discrete negative emotions developed for simultaneous emotion classification (fear, anger, sadness)^53^. Bayes factors were used to quantify evidence for the absence of effects.

**Fig. 2:**
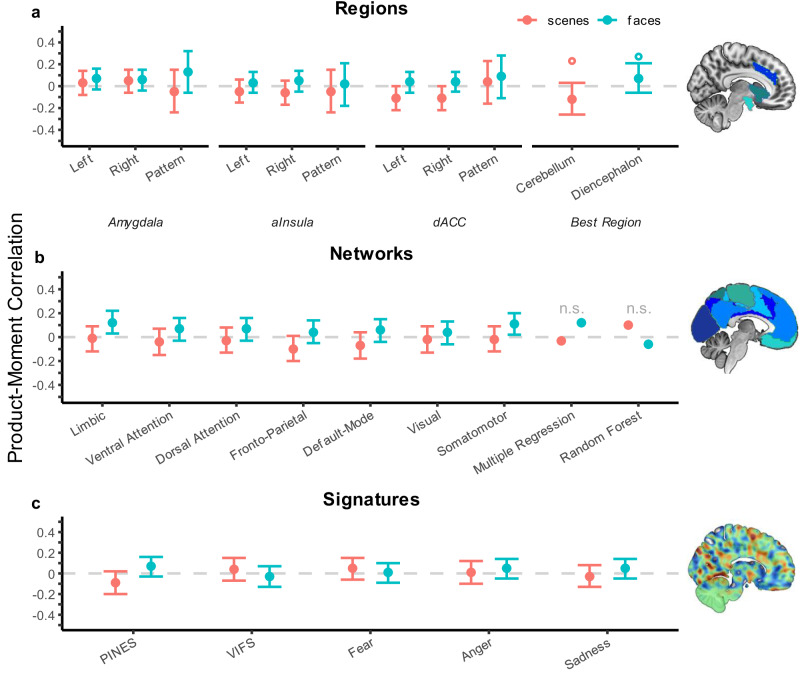
Associations between neuroticism and literature-based neural measures. Pearson’s correlations between neuroticism and individual differences in brain measures for the scenes task (*N* = *338;* red estimates*)* and faces task (*N* = 424; blue estimates) across three neural levels: (**a**) Regions, (**b**) networks, and (**c**) validated multivariate neural signatures. Error bars represent 95% confidence intervals. For region sub-models, “pattern” refers to machine learning models limited to these regions, with estimates shown for the hold-out sample (*N*_Scenes_ = 102; *N*_Faces_ = 101). For the best region approach, hollow circles indicate the point estimates for the region with the largest effect size in the split-half discovery sample (*N*_Scenes_ = 169; *N*_Faces_ = 212). The estimates with error bars indicate results for the split-half validation sample (*N*_Scenes_ = 169; *N*_Faces_ = 212). For the network approach, the variance explained by the multiple regression was tested for significance with an overall *F*-test (uncorrected), while the random forest was evaluated with one-sided tests for significant cross-validated correlations between predicted and actual values based on a permutation test (uncorrected; 1000 iterations). While two single network confidence intervals excluded 0 in the faces task, both did not survive the preregistered correction for 7 network tests; Limbic: uncorrected *p* = 0.013, Holm-adjusted *p* = 0.094; Somatomotor: uncorrected *p* = 0.023, Holm-adjusted *p* = 0.141. For exact estimates see Table S1. dACC = dorsal anterior cingulate cortex, PINES = Picture Induced Negative Emotion Signature, VIFS = Visually Induced Fear Signature.

Overall, there were no clear associations between neuroticism and responses in any literature-based brain measure across regions, resting-state networks, and affective neural signatures (Fig. 2). Most confidence intervals ruled out correlations smaller than |*r* | = 0.20 and even uncorrected Bayes factors mostly favored the null hypothesis over the existence of a true effect with odds above *BF*_01_ = 10 (*BF*_01_ = null hypothesis in the numerator; higher values are expected after multiplicity correction^54^). For precise point estimates, confidence intervals, and Bayes factors see Table S1.

Coincidentally, we observed that task-based responses of all seven networks were very highly correlated on a between-person level (scenes: average *r* = 0.68, range = [0.48–0.84]; faces: average *r* = 0.67, range = [0.48–0.88]). Hence, a person with larger responses in one network also strongly tended to have larger responses in other networks, compared to other people. This demonstrates the extremely limited specificity of task-related responses in these widely used networks for comparisons between individuals. Similar observations have been made for distinct brain regions across a large number of datasets^55^.

In sum—using Bayesian inference, confidence intervals, and cross-validation—we found substantial evidence against meaningful literature-based associations between neuroticism and common brain measures on a neural level of regions, networks, and well-validated affective signatures. Post-hoc correlations between literature-based neural measures and all negative affectivity constructs used in the multiverse analysis are shown in Figs. S1/S2.

### Whole-brain machine learning identifies a stress vulnerability pattern

**Fig. 3:**
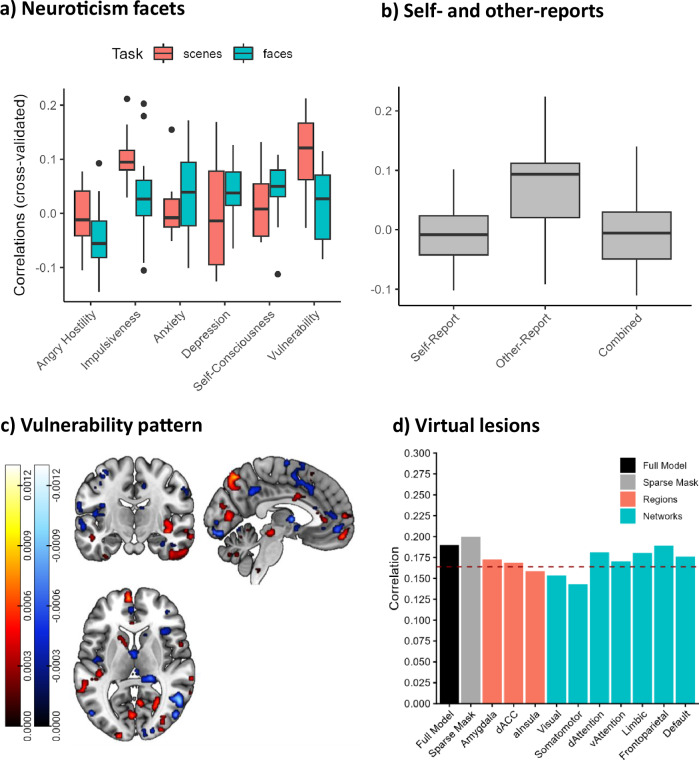
Machine learning prediction of neuroticism facets. **a** Cross-validated correlations in the training data as a function of neuroticism facet and task (18 models per boxplot; estimates for the scenes task are shown in red, estimates for the faces task are shown in blue). **b** Same outcome as a function of self-reports vs reports of close informants vs the combination of both (36 models per boxplot; see methods section). Includes accuracies from all preregistered models. Boxes represent data within the first and third quartile around the median. Whiskers represent the largest observation within 1.5 times the interquartile range, as is standard for boxplots. **c** Significant regression weights for the stress vulnerability pattern in the training data (*N* = 236) after correction at α = .05 and minimum cluster size of 50 (positive weights in red, negative weights in blue). **d** Correlations between brain pattern and hold-out data (*N* = 102) after removing single regions and networks. The dashed red line indicates the significance threshold at α = 0.05 for a one-sided test. “Sparse mask” represents the performance of the full model after applying a more conservative gray matter mask, which decreases the occurrence of artifactual voxels but also has a higher probability to miss relevant voxels. Brain regions are shown in red; large-scale networks are shown in blue. dACC = dorsal anterior cingulate cortex, aInsula = anterior insula, v/dAttention = ventral/dorsal attention network.

In a first stage, twelve partial least squares (PLS) models were used to predict neuroticism in the training sample (Fig. 1c). None of these machine learning models had positive cross-validated correlations with neuroticism (best model: *r* = 0.00). In a second stage, we expanded the model space to 252 models, now also including neuroticism sub-facets and informant ratings of neuroticism as additional outcome constructs as well as support vector regression (SVR) as an additional algorithm (Fig. 1c). Here, the best model predicted the neuroticism facet *stress vulnerability* at *r* = 0.21 (*p* = 0.009; one-sided permutation test) from the scenes task and the contrast [negative – neutral] using SVR and image-wise centering. In the hold-out sample, this correlation was still statistically significant (one-sided): *r*(100) = 0.19, *p* = 0.028, *ΒF*_10_ = 2.53, 95% Confidence Intervals [−0.00, 0.37]. Thus, the whole-brain prediction model reliably generalized to new participants, indicating a robust brain–trait mapping for stress vulnerability and a first step in determining a potential neural marker. Neuroticism, in turn, could not be decoded from responses in the two tasks. Notably, the detected correlation corresponds to the median correlation of meta-analytic individual difference associations across research fields^56^. This highlights that the often extremely high effect sizes from within-person fMRI decoding might not be a suitable frame of reference to evaluate the relevance of between-person effects. For comparisons between predictive accuracies for different constructs and tasks see Fig. 3a/b. The stress vulnerability pattern was further tested concerning its psychological and neurobiological interpretability.

### Reliability and psychological interpretation of the stress vulnerability pattern

A useful personalized biological measure must be both reliable and valid. The stress vulnerability pattern showed high split-half reliability (*r* = 0.83, Spearman–Brown corrected), especially compared to the amygdala response (*r* = 0.29). Construct validity was assessed by correlating leave-one-out cross-validated predictions with trait negative affect (convergent validity) and with trait positive affect and extraversion (discriminant validity). Pattern–score correlations were *r* = 0.25 with stress vulnerability, *r* = 0.13 with negative affect, and near zero with positive affect (*r* = 0.01) and extraversion (*r* = 0.00), providing evidence for convergent and discriminant validity (i.e., positive correlations to related constructs and negligible correlation to conceptually distinct constructs).

To facilitate the psychological interpretation of the observed neural activation pattern, we conducted automated meta-analytic decoding using Neurosynth^57^, which quantifies the association between whole-brain activation maps and large-scale term-based meta-analytic patterns derived from the neuroimaging literature (e.g., “pain”, “fear”, “motoric”). Neurosynth decoding against 50 topic maps from 11,406 fMRI studies indicated that the stress vulnerability pattern has the largest similarity with the broad topic “stimulation” (r = 0.26). Hence, the neural pattern might reflect a broad, non-specific response profile to general external stimulation that is not limited to affective or visual processing. Similarly, spatial correlations between the stress vulnerability pattern and the five literature-based affective signatures were all <|r | = 0.02, indicating the stress vulnerability signature captures something distinct from momentary emotional states.

### The stress vulnerability pattern is distributed across neural systems

No voxel-wise regression weights of the stress vulnerability pattern survived FDR correction (q = 0.05). For descriptive purposes, we applied a lenient threshold (α = 0.05, ≥50 voxels) used in prior work on the same dataset^58^, revealing ~13,000 significant voxels spanning 88 of 489 brain regions across all seven resting-state networks, plus brainstem, cerebellum, and basal ganglia (Fig. 3c; Table S2–3). Some activations slightly outside gray matter might reflect artifacts. Applying a stricter mask removed them and marginally increased performance (Δr = 0.01).

Removing single regions or networks with virtual lesion analyses^59^ did not meaningfully reduce hold-out performance, with the largest performance drop of Δr = 0.05/0.04 for the somatomotor and visual network, respectively Fig. 3d. This indicates that stress vulnerability prediction depended mainly on distributed rather than localized information, with highest emphasis on perception-action-related rather than typical affective networks.

### Image centering indicates limited literature-based neural associations with stress vulnerability

**Fig. 4:**
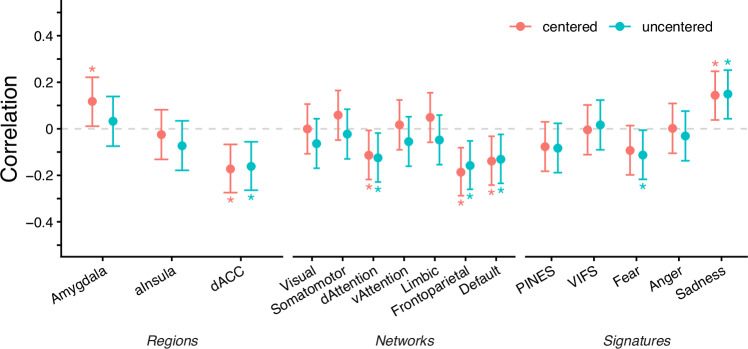
Correlations between stress vulnerability and literature-based neural measures. Correlations are shown with (red) versus without (blue) image-wise centering in the scenes task (*N* = *338)* for the contrast [negative – neutral]. Error bars represent 95% confidence intervals. Asterisks indicate significant Pearson’s correlations (two-sided; uncorrected). Exact statistics are given in Table S4. dACC = dorsal anterior cingulate cortex, aInsula = anterior insula, v/dAttention = ventral/dorsal attention network, PINES = Picture Induced Negative Emotion Signature, VIFS = Visually Induced Fear Signature.

Based on predictive success for the stress vulnerability facet in the scenes task, we explored associations with literature-based neural targets using the same facet, contrast, and task. There was a small significant correlation between stress vulnerability and amygdala responses (uncorrected), in agreement with common theories, but only when images were centered, as in the corresponding machine learning model (Fig. 4). Centering removes individual differences in whole-brain responses, which might be a major confound, as indicated by the extremely high inter-network correlations we observed. For neuroticism, centering did not lead to a significant correlation with amygdala responses, further supporting the absence of a signal for this broad construct in the used tasks (*r*(336) = 0.08, 95% Confidence Intervals = [−0.02, 0.19], *ΒF*_01_ = 6.47, *p* = 0.111). A comprehensive depiction of correlations between literature-based neural measures and all negative affectivity constructs used in the multiverse analyses are shown in Figs. S1/S2 with respect to centering choice.

In contrast, dACC reactivity was associated with *lower* stress vulnerability, contrary to the theory-guided direction, and was not affected by centering. Similar results were seen for the dorsal attention, frontoparietal, and default mode network. This robustness to centering was not explained by higher correlations of global signal responses with the three networks and the dACC. Rather, this lack of change is an algebraically plausible case given the covariance structure between stress vulnerability, global signal responses, and the four uncentered neural measures. Global signal responsiveness was overall slightly negatively correlated with stress vulnerability, speaking against it being due to higher arousal in people with higher stress vulnerability (*r*(336) = −0.08, 95% Confidence Intervals = [−0.19, 0.02], *p* = 0.123). Lastly, there was increased reactivity of the sadness pattern in participants with higher stress vulnerability. Overall, this represents post-hoc evidence that some region-, network-, or signature-based measures might still warrant focused investigation for specific trait facets, especially with certain preprocessing choices like the removal of whole-brain responses.

### Predictability of dynamic affective states

We tested how the predictability of individual differences in negative affective traits compares to the predictability of negative affective *states* based on dynamic self-reports collected during the scenes task. Using the same procedure as for the stress vulnerability pattern, the cross-validated within-person correlations averaged *r* = 0.88 (*SD* = 0.14; see supplemental methods). This means that when analyzing data from the same individual across different time points or conditions, predictive brain patterns can exhibit a strong, consistent relationship with their momentary self-reported affect (Fig. S3). Such high within-person correlations replicate previous findings on this dataset^21^ (r = 0.85) and highlight the predictive gap between within-person and between-person prediction of affective constructs. Specifically, while neural patterns can robustly track fluctuations in an individual’s affective states over time, predicting stable individual differences (i.e., between-person effects) is substantially more challenging (*r* ≤ 0.30; see next section). As Fig. S3 shows, this might be due to participants having considerably different offsets in their average global brain responses regardless of stimulus intensity.

### Exploratory multiverse analysis

**Fig. 5:**
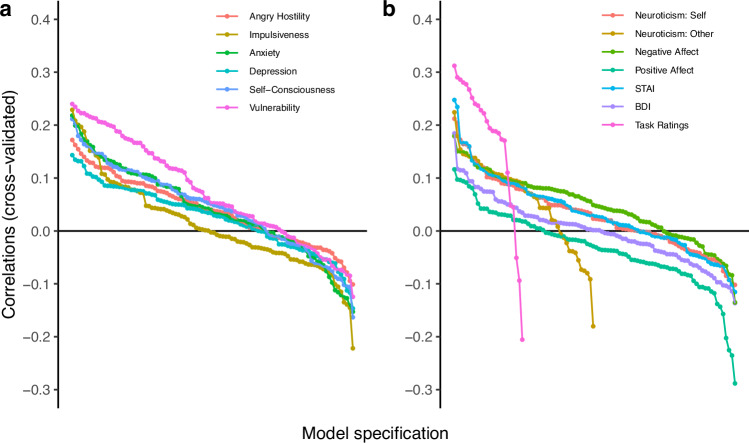
Multiverse analyses show selective predictability of negative affectivity constructs. Cross-validated correlations of all fitted models, separated by (**a**) neuroticism facets and (**b**) other affective traits. Models are ordered based on their effect size along the *x*-axis. “Task ratings” refers to a participants’ average negative affective self-ratings during the scenes task. There were no similar ratings for the faces task, leading to a smaller number of models. Neuroticism scores provided by informants (“Neuroticism: Other”) were only collected in a subset of 165 individuals. Therefore, we only fitted these models with all available data, instead of additionally using training data only (Fig. 1c; Data choice), leading to a reduced number of models. STAI = State-Trait Anxiety Inventory, BDI = Beck Depression Inventory.

We extended the range of trait constructs and preprocessing choices for machine learning models to a more extensive multiverse analysis of 1176 models to inform the predictability of negative affectivity traits more broadly (see Fig. 1c for all included modeling choices). Figure 5 shows the cross-validated correlations of all models, separately for the different constructs serving as outcomes. Qualitatively, three important observations can be made. Firstly, most neuroticism facets had predominantly positive cross-validated correlations, indicating a signal in the data, except for impulsiveness (Fig. 5a). Secondly, stress vulnerability had the strongest associations among neuroticism facets in a large range of models, supporting the results of the preregistered analyses. Thirdly, a comparison of different negative affectivity traits largely conforms to a face validity gradient (Fig. 5b): The highest correlations were achieved for average task-based affective ratings, followed by negative affective dispositions (STAI, neuroticism other-reports, and neuroticism-self-reports), while positive affect had the lowest correlations, with depressive symptoms in between.

**Fig. 6:**
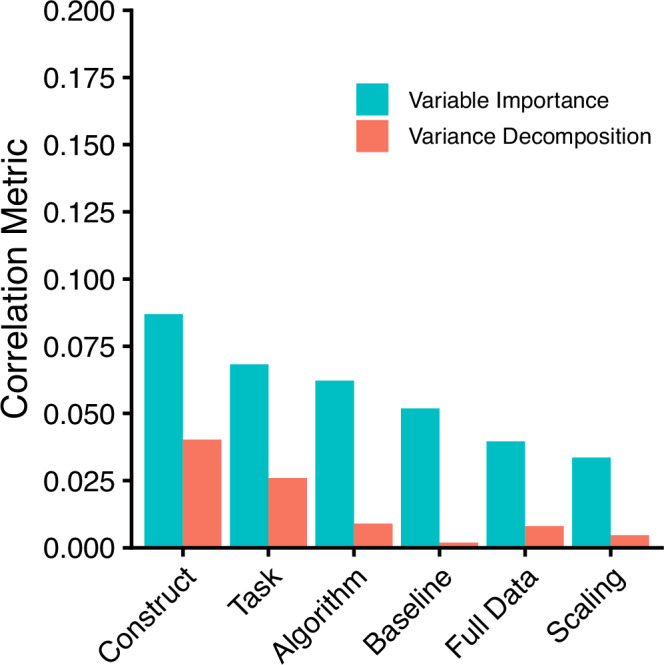
Construct and task choice explain most variation in predictive accuracy. Design factors and cross-validated correlations. Blue bars show variable importance when predicting correlations from design factors using random forest regression. It reflects the drop in the accuracy when a given predictor is randomly shuffled in the metric of root mean squared error (i.e., average deviation between predicted and actual correlations). Red bars show standard deviations from a linear mixed model variance decomposition (i.e., how much correlations differ between design choices within a given factor). Levels of design factors are shown in Fig. 1c.

A random forest regression on design factors explained 22% variance in the size of correlations. The most important design factors were construct choice and task choice, as confirmed by a variance decomposition (Fig. 6). The scenes task provided overall better results than the faces task, potentially as it relates more closely to the experience rather than perception of emotion in others. This confirms that the two common fMRI tasks do not contain information on any trait, but that matching tasks and constructs is essential. Moreover, we found that PLS, PCR, and SVR worked similarly well, with worse performance for random forest regression. The observation that centering choice had only a minor effect on accuracy likely indicates that these three algorithms implicitly account for global signal confounding. This is demonstrated in Figs. S4/S5. Furthermore, using the full sample improved performance, supporting that higher accuracy might be achieved with still larger samples. Lastly, in post-hoc analyses we observed that the prediction of stress vulnerability was better when using only highly intense trials (rating = 5, *r* = 0.05) than moderately intense trials (rating = 3, *r* = 0.00), although both models were well-below the results for averaging all trials. This might point towards the relevance of both the number of trials and the intensity of stimuli.

As there are a large number of design factor combinations which might be of interest for closer inspection, we provide an online application with an accessible point-and-click user interface to interactively explore these results: https://msicorello.shinyapps.io/ndesignshinyapp/.

## Discussion

Negative affectivity is a key dimension of psychopathology, general well-being, and personality, yet its neural basis remains elusive. Here, we conducted one of the most comprehensive investigations to date into the functional neurobiology of negative affectivity, combining preregistered and exploratory analyses across the two most widely used affective fMRI tasks and a broad range of trait constructs.

Large parts of the fMRI literature have treated a context-general hyperresponsiveness to negative stimuli in putative affective networks as neural markers of negative affectivity. Our findings instead support a construct–assay alignment principle: robust brain–trait associations emerge when the experimental task selectively engages the relevant neural computations for a specific affective construct. This was most clearly demonstrated by the machine learning multiverse analysis, which indicated construct and task choice as the most important factors influencing the ability to decode complex traits from neural responses. Construct decodability followed a face validity gradient from more task-related constructs (e.g., task-evoked ratings, stress vulnerability) to relatively task-unrelated constructs (e.g., BDI, positive affect). Together with the preregistered and independently validated detection of a neural signature for stress vulnerability, these findings highlight a concrete path forward in a research field that has been confronted with severe challenges in producing robust and meaningful effects^37,60–62^: A more precise construct delineation and aligned task selection.

The idea of a context-general neural hyperresponsiveness to negative stimuli has been particularly influential for biological theories of neuroticism, a major domain of personality and dimensional psychopathology. The amygdala and salience network, but also anterior insula and dACC, have been core targets to explain this broad variant of negative affectivity^5,12,13^. In comprehensive theory-guided preregistered analyses, we found considerable evidence against these assumptions, supported by Bayes factors. Neither responses in regions, canonical resting-state networks, validated affective neural signatures, or data-driven machine learning models showed meaningful associations with neuroticism. Hence, our results speak against the widespread idea of a context-general amygdala hyper-responsiveness to negative stimuli underlying neuroticism and against simple psychological interpretations of person-specific responsivity in these regions and networks. This finding has implications for clinical research, due to (1) the fact that the two tasks we studied are frequently used as indicators of negative affectivity and (2) the strong overlap between neuroticism, negative affectivity in the DSM-5, and negative valence system measures of the Research Domain Criteria^4^.

In contrast to broad neuroticism, stress vulnerability was robustly predictable from task-evoked brain responses and replicated out-of-sample. Virtual lesion analyses indicated that prediction relied most strongly on visual and somatomotor systems, consistent with a distributed perception–action profile rather than localized “affective hotspot” reactivity. In line with this, the pattern showed little dependence on canonical affective regions or networks and was largely orthogonal to validated neural signatures of momentary affect, suggesting that it does not primarily indicate affect intensity. This agrees with the observation that task-based affective reports were unrelated to all negative affectivity constructs (Table 2), as previously observed for clinical negative affectivity^35^. Instead, converging evidence implicates the occipital visual cortex in encoding emotionally diagnostic stimulus information and somatomotor systems in embodied, action-oriented representations of emotional meaning^42,63–66^. Together, these findings are consistent with the possibility that individuals high versus low in stress vulnerability differ less in core affective feelings elicited by non-personalized laboratory stimuli than in how they construe and prepare to respond to aversive events—i.e., in the perception- and action-related relevance assigned to negative scenes. Such individual differences in affective conceptualizations might be more generalizable across experimental tasks, while an additional contribution of other regions could still emerge if personally relevant stressors are ascribed meaning^67^.

The stress vulnerability signature also shows promise as a candidate neural marker. Besides replication in the hold-out sample, it exhibited good split-half reliability and evidence for construct validity. Predictive accuracy was modest (cross-validated r ≈0.20), but this magnitude is consistent with the median correlational effects observed in individual-differences research^56^, which provides a realistic expectation for transmodal brain–questionnaire associations.

Our preregistered machine learning analyses highlight that not all facets of negative affectivity are equally decodable from negative affective scenes and faces. Anticipating that these tasks are less related to more approach-oriented sub-facets of neuroticism (impulsiveness, angry hostility), we preregistered the qualitative expectation of higher decodability for withdrawal-related facets (stress vulnerability, depression, anxiety, self-consciousness). Still, the multiverse analysis showed only a predictive advantage of stress vulnerability, not the remaining withdrawal-facets, which was also only apparent for the scenes task. This is plausible as the psychological definition of stress vulnerability mirrors the scenes task: How individuals respond to negative events. In contrast, depression is more focused on prolonged affective states and anxiety on the anticipation of threat. Therefore, qualitatively different tasks might better target those facets, like ruminative state switching^68^ and threat-of-shock^69^, respectively. We nevertheless focused on negative scenes and face viewing because they are the most widely used affective fMRI tasks and are often treated as broadly applicable probes of ‘general’ negative responsivity, consistent with biological theories of negative affectivity^3,12,14,35^. Using these canonical tasks therefore provides a stringent and maximally field-relevant test of the context-generality assumption, which still underlies current large-scale studies^70^. At the same time, our findings do not exclude that other tasks could yield more accurate predictions of negative affectivity facets or even broad neuroticism. Identifying robust facet-selective task contexts remains an open methodological need for the field^1,71,72^. Importantly, one possibility is that broad negative affectivity is “the sum” of facet-specific mechanisms that are each most visible in different, facet-selective task contexts. If so, higher-order traits like neuroticism may only yield coherent neural correlates when aggregating across a battery of such tasks, also clarifying which psychological constructs have a consistent neurobiological counterpart.

Importantly, the null findings for canonical affective systems do not invalidate their role in dynamic affective *states*, as they reliably respond to the tasks employed in the present study^21,23,73^. Rather, their explanatory capability for negative affectivity *traits* is subject to scientific boundary conditions, comprising the discussed assumption of relative context-generality across tasks and an appropriate analytical strategy. In that regard, we found that individual differences in global signal responses might confound associations with single regions. We made similar observations in a parallel emotion regulation consortium across datasets, further emphasizing the generalizability of this methodological issue^55^. Specific machine learning algorithms like partial least squares implicitly remove such effects, further supporting their predictive superiority. After controlling global signal effects within exploratory analyses, amygdala responses in the scenes task were related to stress vulnerability with a small effect size, but unrelated to neuroticism as well as most other negative affectivity constructs in exploratory analyses. Similar positive associations were observed for the affective sadness signature. A potential explanation might be that pictures of threatening events (e.g., people with wounds) elicit emotions related to sadness rather than fear, when viewed in a safe laboratory environment. In contrast, several regions and networks showed stable negative associations with stress vulnerability, including the dACC, dorsal attention, frontoparietal, and default mode network. Especially the dACC finding is contrary to the theoretical assumption of higher salience-related responsiveness people with high negative affectivity. In principle, lower dACC, dorsal attention, and frontoparietal network recruitment could indicate deficits in externally-oriented executive function accompanied with sustained stimulus engagement, as indicated by lower default mode network deactivation. Still, region and network labels are coarse descriptors and each network supports multiple computations with context-dependent engagement.

Two limitations should be noted. Firstly, although our predictive model for stress vulnerability replicated in a hold-out sample, its generalizability to other designs and populations remains to be tested. Especially for clinical samples phenomena like trait dissociation can be important confounds, interfering with neuro-affective reactivity^74^. While our sample exhibited a representative range of neuroticism scores^47^, future studies may benefit from oversampling participants with clinically elevated affective problems. For such efforts, we openly provide the stress vulnerability pattern in our online repository. Secondly, there might be physiological confounds which improved the predictive utility of our pattern. On the one hand, such confounds are not easily controlled, as there is a complex bi-directional relation between neural and physiological data (e.g., heart rate increases due to affect-related neural processing, but can also contribute to noise in the fMRI BOLD signal). On the other hand, it might still be of interest to test the incremental value of our pattern above peripheral physiological measures in future studies.

Together, these findings yield several concrete recommendations for future research on the neurobiology of negative affectivity: (a) tasks should be explicitly designed for specific psychological constructs; (b) distributed whole-brain patterns offer more robust predictive power than region-focused models; (c) preprocessing strategies should address global signal biases; (d) realistic maximal effect size expectations for trait prediction should be anchored around r = .20–.30; and (e) samples should cover the full dimensional spectrum of trait variation.

In conclusion, our results challenge the dominant assumption that broad negative affectivity—especially neuroticism—maps onto a context-general hyperresponsiveness of canonical “affective” regions and networks during negative face and scene viewing. Instead, they support a construct–assay alignment principle: reliable brain–trait associations emerge when tasks elicit computations that closely match the psychological content of the target construct. Within this framework, stress vulnerability—but not neuroticism—showed replicable, distributed whole-brain predictability, pointing to individual differences in the conceptual perception–action relevance assigned to aversive events rather than generalized affective intensity. More broadly, these findings might suggest that progress in the neurobiology of negative affectivity will depend less on simply increasing sample size with generic affective tasks, and more on tighter construct delineation, process-selective task batteries, and analytical pipelines that address global signal biases while maintaining realistic expectations for trait-level effect sizes.

## Methods

The timestamped preregistration (October 2020) of aims, hypotheses, and statistical analyses as well as data, analysis code, and trained model patterns can be downloaded via: https://github.com/MaurizioSicorello/NeuroSquare_repo/blob/main/Preregistration.docx.

### Participants

The data comprised baseline assessments from up to 424 participants from the Adult Health and Behavior project (phase 2; AHAB-2) and the Pittsburgh Imaging Project (PIP). Therefore, this Methods section partially overlaps with details from previous publications (especially one study^58^, which used identical MRI preprocessing). All 424 participants provided data for the faces task, while only a subset of 338 participants provided data for the scenes task. Sample characteristics are provided in Table 1. Given the universality of the tested theories, and the absence of a strong theoretical basis for sex- or gender-specific hypotheses, no such hypotheses were preregistered and no sex- or gender-stratified analyses were conducted. All participants provided informed consent. Participants were compensated $350.00 in AHAB-2 and $175.00 in PIP for completing all protocol visits. The University of Pittsburgh Human Research Protection Office granted approval for AHAB-2 (Protocol ID: 07040037) and PIP (07110287), as well as their aggregation to create a common data registry (19030174).

### Psychological measures

Neuroticism and extraversion were measured with self-reports from the full NEO-PI-R^75^, and “other-reports” from up to two close informants in AHAB-2 using the NEO-FFI^47^ (spouses/partners, first-degree relatives, close friends and co-workers). Positive and negative affect were measured using the Positive and Negative Affect Schedule^76^. Trait Anxiety was measured with the State-Trait Anxiety Inventory^77^. Dimensional depression severity was measured with the Beck Depression Inventory^78^. Individual differences in task-based responsivity to negative stimuli were measured as the person-wise average difference in self-reported momentary affect between negative and neutral experimental trials, based on a single-item 5-point Likert scale (see task description for further information). Descriptive statistics and correlations are shown in Table 2. The main outcome, neuroticism, has an estimated internal consistency of *r* = 0.93 and test-retest reliability over three months of *r* = 0.79 (Costa & McCrae, 1992). Internal consistency of subfacets is slightly lower around *r* = 0.80.

### Experimental tasks

Schematics of task structures are depicted in Fig. 1b for both tasks.

#### Scenes task

Subgroups of AHAB-2 and PIP participants completed an affective processing and responding task, which has been detailed previously^79^. Participants saw 30 unpleasant and 15 neutral IAPS images^80^. Participants were first trained and then instructed to (i) ‘Look’ and attend to images or (ii) ‘Decrease’ and change their thinking about the image to feel less negative. Trials were comprised of a 2 s cue (‘Look’ or ‘Decrease’), followed by a 7 s IAPS image presentation. After image viewing, participants rated their emotional state (‘How negative do you feel?’) on a 5-point Likert-type scale in a 4 s rating period (1 = neutral, 5 = strongly negative). A variable (1–3 s) rest period followed each rating period. The entire task duration was 11 min and 16 s (15 ‘Look neutral’ trials; 15 ‘Look negative’ trials; 15 ‘Decrease negative’ trials). Images were presented such that no more than two identical trial types (‘Look negative’ or ‘Decrease negative’) were consecutive and no more than four unpleasant images were consecutive. Given the focus of this report (and for comparability to the facial expression tasks involving the passive viewing of affective stimuli), only ‘Look neutral’ and ‘Look negative’ trials were analyzed. Across AHAB-2 and PIP, unpleasant images for ‘Look negative’ trials overlapped by 87% (13/15 shared images) and neutral images by 13% (2/15 shared images). E-Prime software (Psychology Software Tools, Sharpsburg, PA) was used to present stimuli and record behavioral responses in both the faces and scenes tasks (for a list of image stimuli, see ref. ^58^).

#### Faces task

In this task, participants completed four blocks of a facial expression-matching-to-sample condition, which was interleaved with five blocks of a shape-matching (sensorimotor) control condition. In the facial expression matching condition, participants saw three same-sex faces in an array for each trial, all expressing either fear or anger. Participants chose one of the two faces at bottom that was identical to a center target at top. Each block consisted of six trials (three fear, three anger; three all-male, three all-female), and each trial lasted for 4 s (1.5, 3.5 and 5.5 s variable inter-trial interval; ITI). All images were in black and white and were drawn from the Pictures of Facial Affect (PFA) stimulus set^81^. In the control condition, participants also matched-to-sample but instead used images of circles, vertical ellipses and horizontal ellipses. Each trio of shapes was shown for 4 s with a 2 s ITI. The total task length was 6 min and 36 seconds, including 6 s for initial magnetic equilibration. Similar tasks have been used frequently in the study of emotion processing and psychopathology, including the large-scale UK Biobank and ABCD studies^82,83^.

### MRI data acquisition and analysis

Functional blood-oxygen-level-dependent (BOLD) images from AHAB-2 and PIP participants were collected on the same 3 Tesla Trio TIM whole-body scanner (Siemens, Erlangen, Germany), equipped with a 12-channel head coil. Further details on data acquisition and pre-processing can be found in the supplements. Univariate general linear models (GLMs) were estimated to compute condition or event contrast maps that were later used for prediction analysis. Stimulus time series were convolved with the default hemodynamic response function in SPM12. For the facial expression matching task, these regressors modeled blocks of face- and shape-matching conditions using boxcar functions. For the scenes task, these regressors modeled events of the trial (i.e., cue, picture, rating period, rest). Each GLM additionally included six motion regressors of no interest and a high-pass temporal filter (128 s) to correct for low frequency drift. Group mean contrasts were estimated using restricted maximum likelihood, as implemented in the RobustWLS toolbox, v4.0^84^. These included the ‘Faces vs Shapes’ contrast for the facial expression-matching tasks and ‘Look negative vs Look neutral’ contrast for the scenes task.

### Data exclusion

One participant in the faces task was a multivariate outlier according to Bonferroni-Holm-corrected Mahalanobis distance and therefore excluded from analyses on that task. The sample size reported throughout this manuscript reflects the effective (i.e., final) sample size.

### Statistical analysis

#### Power analysis of hold-out samples

A hold-out sample of *N* = 102 for the scenes task and of *N* = 101 for the faces task was drawn for the evaluation of machine learning models. Training and hold-out samples were stratified for neuroticism using the caret package in R (v4.3.2) to make the two subsamples comparable. The discrepancy of one participant was due to the different number of participants for the two tasks and rounding in the stratification procedure. This sample has a power of 0.90 to detect a true correlation between pattern and neuroticism of r ≈ 0.30 in a one-tailed test, which is at the 75% percentile of questionnaire-behavior correlations in individual difference research^56^.

#### Literature-based approaches

For the literature-based approaches, neural activity was assessed in the contrasts for negative vs neutral scenes and negative faces vs shapes. An overview of models is illustrated in Fig. 1a.

##### Regions

Multiple atlases were used to parcellate the brain into 489 anatomic regions taken from the canlab toolbox^85,86^. From these, masks for the amygdala, anterior insula, and dACC were built. We conducted three region-based tests, which vary in complexity. First, correlations between average signal in these regions and neuroticism scores was calculated on the full data, separately for the two hemispheres and Bonferroni-Holm corrected for six tests. Secondly, we predicted neuroticism scores from voxel-wise activity in each region in the training sample using cross-validated partial least squares regression and evaluated performance in the hold-out sample. We used the same procedure as described in the machine learning section below. This was done to account for the fact that using only the average signal of these regions might be too coarse. Thirdly, we extended our analysis to regions beyond the three main regions. We split the sample in half, stratified for neuroticism, picked the region with the strongest correlation in the first half and tested its correlation in the second half.

##### Networks

A canonical network parcellation was used to extract the average task-based responses in seven resting state networks^51^: Limbic, Ventral Attention (Salience Network), Dorsal Attention, Fronto-Parietal, Default-Mode, Visual, and Somatomotor. Again, we used three different approaches, which differ in complexity. First, we correlated this signal with neuroticism in the full sample to test the simple hypothesis that one of these networks predominantly underlies negative affectivity, correcting for seven tests using the Bonferroni-Holm procedure (the preregistration stated an incorrect correction for eight tests). Then, we predicted neuroticism from average activity in all seven networks using multiple regression to test whether neuroticism is represented by a linear combination of network activities. Last, we repeated this predictive model with random forest regression to allow for non-linear effects and interactions between networks. For the latter model, performance was evaluated using the out-of-bag samples (a form of cross-validation) and a permutation test with 1000 random permutations of neuroticism scores.

##### Signatures

We calculated the neural expression of five well-validated neural signatures for negative affect (PINES)^21^, fear (VIFS)^23^, and three discrete negative emotions^53^ by taking the dot product of model weights and person-wise functional images. The VIFS was published after our preregistration and, therefore, added post-hoc. Moreover, we preregistered an analysis combining the three discrete emotions into one factor score for “neural negative affect”, before assessing them separately, but their intercorrelations were too low to make this a sensible approach (all r <.12). A Bonferroni-Holm correction for three tests was preregistered for the discrete emotion patterns, but a correction for five tests is also sensible.

#### Data-driven machine learning models

The general preregistered strategy was to (1) split the data into a training and a hold-out sample, (2) predict neuroticism from different models in the training sample using nested cross-validation, (3) retrain the best model on the entire training dataset, and (4) assess performance in the hold-out sample. An overview of all modeling choices can be found in Fig. 1c. Machine learning analyses were performed using the CanlabCore Toolbox^86^ with Matlab R2023b.

In step 1, the hold-out sample was set aside with the procedure described in the power analysis section.

In step 2, we predicted neuroticism in the training sample using Partial Least Squares regression from a small set of 12 “first stage” models (Fig. 1c) with different combinations of task choice (scenes vs faces), baseline choice (neutral scenes or shapes vs implicit baseline), and image-wise scaling choice (z-scoring vs centering vs no scaling). All whole-brain machine learning models were conducted after applying a gray matter mask and z-standardizing voxel-wise values on the sample mean and standard deviation of that voxel. As other studies will have different voxel-wise statistics, predictions from our model will have an offset and can only serve as a correlate of trait measures, rather than predicting exact numeric values. Repeated nested cross-validation (2 × 5 × 5) was used to calculate cross-validated correlations between pattern predictions and outcome scores, with five inner folds for hyperparameter tuning, five outer folds for model evaluation, and two repeats for stability. Pearson’s correlations were used as a performance metric, as the aim was to train a pattern for scale-independent correlates of negative affectivity, which can be used across different studies with different scanning parameters or different questionnaires. We preregistered that if the best-fitting model reaches *p* < 0.05 (one-tailed) in a permutation test, the model is accepted and will be validated in the hold-out sample, again with a one-tailed *p* < .05 and supplemented with a Bayes factor with default low-informative half-cauchy priors for one-sided tests from the Bayes factor package in R^87^. One-tailed tests were used, as only positive correlations indicate successful prediction. If this criterion was not fulfilled, we preregistered a plan to move on to a larger set of 252 “second stage” models (Fig. 1c), comprising several choices of constructs (neuroticism and its six facets) and algorithms (Partial Least Squares [PLS], Principal Component Regression [PCR], and Support Vector Regression [SVR]). We also preregistered the inspection of self- and other-based ratings, as well as their combination. For further information on hyper-parameter estimation and model evaluation see the supplements. The machine learning procedure was tested with a simulated dataset of *N* = 100 and 20 predictive features, implemented as a “test mode” in the main script.

#### Exploratory multiverse analysis

In a post-hoc multiverse analysis, we calculated a larger set of 1176 “third stage” models (Fig. 1c) to assess the effect of different negative affectivity traits and modeling choices on predictive accuracy. Additional constructs included negative affect, positive affect, STAI scores, BDI scores, other-reports for neuroticism, and task-based affective self-ratings collected during the scenes task (person-wise averaged for the negative minus neutral condition). Other modeling choices included using the full data (training plus hold-out) to assess the effects of sample size and using random forest regression as an algorithm which accommodates nonlinear and interaction effects. For random forest regression, principal component analysis was used to reduce the feature space to the number of participants in the training data.

The impact of design choices was assessed by predicting brain-outcome-correlations from design factors using random forest regression with 1000 bagged trees (2 × 5 = 10 correlations per model). The relevance of single design predictors was quantified with permutation-based variable importance. These analyses were conducted with the cforest package in R. Moreover, we performed a variance decomposition to quantify which design factor is associated with the largest differences in cross-validated correlations. These analyses were only performed with descriptive rather than inferential statistics, as the latter might be incorrect due to complex dependencies in the data^88^.

As this number of models was already very large and computationally intensive, we omitted the additional preregistered choices of image-type (beta-map versus t-map), construct modeling (sum scores versus factor scores), and the neuroticism spectra withdrawal and volatility in all analyses, as we believed these choices to have little impact on performance (beta-images and t-images are highly correlated, all information of neuroticism spectra is contained in the more fine-grained facets, and factor scores, in contrast to latently modeled variables, also contain measurement error and are extremely highly correlated with sum scores^89,90^).

### Reporting summary

Further information on research design is available in the Nature Portfolio Reporting Summary linked to this article.

## Supplementary information

Supplementary Information

Reporting Summary

Transparent Peer Review file

## Data Availability

Deidentified psychological assessment data and neuroimaging data can be accessed, respectively, via https://github.com/MaurizioSicorello/NeuroSquare_repo^91^ and https://identifiers.org/neurovault.collection:5802.

## Code availability

Custom code is available on: https://github.com/MaurizioSicorello/NeuroSquare_repo^91^.

## References

[1] Brandt, A. & Mueller, E. M. Negative affect related traits and the chasm between self-report and neuroscience. *Curr. Opin. Behav. Sci.* **43**, 216–223 (2022).

[2] DeYoung, C. G. et al. Personality neuroscience: an emerging field with bright prospects. *Personal. Sci.* **3**, e7269 (2022). 36250039 10.5964/ps.7269 PMC9561792

[3] Eysenck, H. J. *The Biological Basis of Personality* (Spring-field, Ill., Thomas, 1967).

[4] Gore, W. L. & Widiger, T. A. Negative emotionality across diagnostic models: RDoC, DSM-5 section III, and FFM. *Personal. Disord. Theory Res. Treat.* **9**, 155–164 (2018). 10.1037/per0000273 29578748

[5] Shackman, A. J. et al. Dispositional negativity: an integrative psychological and neurobiological perspective. *Psychol. Bull.* **142**, 1275–1314 (2016). 27732016 10.1037/bul0000073 PMC5118170

[6] Wright, A. G. C. & Simms, L. J. A metastructural model of mental disorders and pathological personality traits. *Psychol. Med.* **45**, 2309–2319 (2015). 25903065 10.1017/S0033291715000252 PMC4498970

[7] Kotov, R. et al. The hierarchical taxonomy of psychopathology (HiTOP): a dimensional alternative to traditional nosologies. *J. Abnorm. Psychol.* **126**, 454–477 (2017). 28333488 10.1037/abn0000258

[8] Cuthbert, B. N. The RDoC framework: facilitating transition from ICD/DSM to dimensional approaches that integrate neuroscience and psychopathology. *World Psychiatry* **13**, 28–35 (2014). 24497240 10.1002/wps.20087 PMC3918011

[9] Kozak, M. J. & Cuthbert, B. N. The NIMH research domain criteria initiative: background, issues, and pragmatics. *Psychophysiology* **53**, 286–297 (2016). 26877115 10.1111/psyp.12518

[10] Fried, E. I. Lack of theory building and testing impedes progress in the factor and network literature. *Psychol. Inq.* **31**, 271–288 (2020).

[11] Seeley, W. W. The salience network: a neural system for perceiving and responding to homeostatic demands. *J. Neurosci.* **39**, 9878–9882 (2019). 31676604 10.1523/JNEUROSCI.1138-17.2019 PMC6978945

[12] Mitchell, R. L. C. & Kumari, V. Hans Eysenck’s interface between the brain and personality: modern evidence on the cognitive neuroscience of personality. *Personal. Individ. Differ.* **103**, 74–81 (2016).

[13] Ormel, J. et al. The biological and psychological basis of neuroticism: current status and future directions. *Neurosci. Biobehav. Rev.* **37**, 59–72 (2013). 23068306 10.1016/j.neubiorev.2012.09.004

[14] Linehan, M. M. *Cognitive-Behavioral Treatment of Borderline Personality Disorder* (Guilford Press, New York, 1993).

[15] Kievit, R. A., Frankenhuis, W. E., Waldorp, L. J. & Borsboom, D. Simpson’s paradox in psychological science: a practical guide. *Front. Psychol*. 10.3389/fpsyg.2013.00513 (2013). 10.3389/fpsyg.2013.00513 PMC3740239 23964259

[16] Rohrer, J. M. & Murayama, K. These are not the effects you are looking for: causality and the within-/between-persons distinction in longitudinal data analysis. *Adv. Methods Pract. Psychol. Sci.* **6**, 25152459221140842 (2023).

[17] Kragel, P. A., Koban, L., Barrett, L. F. & Wager, T. D. Representation, pattern information, and brain signatures: from neurons to neuroimaging. *Neuron* **99**, 257–273 (2018). 30048614 10.1016/j.neuron.2018.06.009 PMC6296466

[18] Cunningham, W. A. & Brosch, T. Motivational salience: amygdala tuning from traits, needs, values, and goals. *Curr. Dir. Psychol. Sci.* **21**, 54–59 (2012).

[19] Lindquist, K. A., Satpute, A. B., Wager, T. D., Weber, J. & Barrett, L. F. The brain basis of positive and negative affect: evidence from a meta-analysis of the human neuroimaging literature. *Cereb. Cortex* **26**, 1910–1922 (2016). 25631056 10.1093/cercor/bhv001 PMC4830281

[20] Uddin, L. Q. Salience processing and insular cortical function and dysfunction. *Nat. Rev. Neurosci.* **16**, 55–61 (2015). 25406711 10.1038/nrn3857

[21] Chang, L. J., Gianaros, P. J., Manuck, S. B., Krishnan, A. & Wager, T. D. A sensitive and specific neural signature for picture-induced negative affect. *PLoS Biol.* **13**, 1–28 (2015). 10.1371/journal.pbio.1002180 PMC4476709 26098873

[22] Krishnan, A. et al. Somatic and vicarious pain are represented by dissociable multivariate brain patterns. *eLife* **5**, e15166 (2016). 27296895 10.7554/eLife.15166 PMC4907690

[23] Zhou, F. et al. A distributed fMRI-based signature for the subjective experience of fear. *Nat. Commun.* **12**, 1–16 (2021). 34789745 10.1038/s41467-021-26977-3 PMC8599690

[24] Fullana, M. A. et al. Fear extinction in the human brain: a meta-analysis of fMRI studies in healthy participants. *Neurosci. Biobehav. Rev.* **88**, 16–25 (2018). 29530516 10.1016/j.neubiorev.2018.03.002

[25] Taschereau-Dumouchel, V., Kawato, M. & Lau, H. Multivoxel pattern analysis reveals dissociations between subjective fear and its physiological correlates. *Mol. Psychiatry*10.1038/s41380-019-0520-3 (2019). 10.1038/s41380-019-0520-3 PMC7515839 31659269

[26] Visser, R. M., Bathelt, J., Scholte, H. S. & Kindt, M. Robust BOLD responses to faces but not to conditioned threat: challenging the amygdala’s reputation in human fear and extinction learning. *J. Neurosci.* **41**, 10278–10292 (2021). 34750227 10.1523/JNEUROSCI.0857-21.2021 PMC8672698

[27] Inman, C. S. et al. Human amygdala stimulation effects on emotion physiology and emotional experience. *Neuropsychologia* 106722 10.1016/j.neuropsychologia.2018.03.019 (2020). 10.1016/j.neuropsychologia.2018.03.019 PMC6139084 29551365

[28] Goodkind, M. et al. Identification of a common neurobiological substrate for mental illness. *JAMA Psychiatry* **72**, 305–315 (2015). 25651064 10.1001/jamapsychiatry.2014.2206 PMC4791058

[29] Schulze, L., Schulze, A., Renneberg, B., Schmahl, C. & Niedtfeld, I. Neural correlates of affective disturbances: a comparative meta-analysis of negative affect processing in borderline personality disorder, major depressive disorder, and posttraumatic stress disorder. *Biol. Psychiatry Cogn. Neurosci. Neuroimaging* **4**, 220–232 (2019). 30581154 10.1016/j.bpsc.2018.11.004

[30] Sha, Z., Wager, T. D., Mechelli, A. & He, Y. Common dysfunction of large-scale neurocognitive networks across psychiatric disorders. *Biol. Psychiatry* **85**, 379–388 (2019). 30612699 10.1016/j.biopsych.2018.11.011

[31] Chen, Y.-W. & Canli, T. “Nothing to see here”: No structural brain differences as a function of the Big Five personality traits from a systematic review and meta-analysis. *Personal. Neurosci.* **5**, e8 (2022). 35991756 10.1017/pen.2021.5 PMC9379932

[32] Lin, J. et al. Neural correlates of neuroticism: a coordinate-based meta-analysis of resting-state functional brain imaging studies. *Neurosci. Biobehav. Rev.* **146**, 105055 (2023). 36681370 10.1016/j.neubiorev.2023.105055

[33] Liu, X. et al. Gray matter structures associated with neuroticism: a meta-analysis of whole-brain voxel-based morphometry studies. *Hum. Brain Mapp.* **42**, 2706–2721 (2021). 33704850 10.1002/hbm.25395 PMC8127153

[34] Mincic, A. M. Neuroanatomical correlates of negative emotionality-related traits: a systematic review and meta-analysis. *Neuropsychologia* **77**, 97–118 (2015). 26265397 10.1016/j.neuropsychologia.2015.08.007

[35] Sicorello, M. & Schmahl, C. Emotion dysregulation in borderline personality disorder: a fronto–limbic imbalance? *Curr. Opin. Psychol.* **37**, 114–120 (2021). 33422855 10.1016/j.copsyc.2020.12.002

[36] Xu, J. et al. Anxious brain networks: a coordinate-based activation likelihood estimation meta-analysis of resting-state functional connectivity studies in anxiety. *Neurosci. Biobehav. Rev.* **96**, 21–30 (2019). 30452934 10.1016/j.neubiorev.2018.11.005

[37] Marek, S. et al. Reproducible brain-wide association studies require thousands of individuals. *Nature* **603**, 654–660 (2022). 35296861 10.1038/s41586-022-04492-9 PMC8991999

[38] Schulz, M.-A., Bzdok, D., Haufe, S., Haynes, J.-D. & Ritter, K. Performance reserves in brain-imaging-based phenotype prediction. *Cell Rep*. **43**, 113597 (2024). 10.1016/j.celrep.2023.113597 PMC11215805 38159275

[39] Bolger, N. & Schilling, E. A. Personality and the problems of everyday life: the role of neuroticism in exposure and reactivity to daily stressors. *J. Pers.* **59**, 355–386 (1991). 1960637 10.1111/j.1467-6494.1991.tb00253.x

[40] Bolger, N. & Zuckerman, A. Personality processes and individual differences: a framework for studying personality in the stress process. *J. Pers. Soc. Psychol.* **69**, 890–902 (1995). 7473036 10.1037//0022-3514.69.5.890

[41] Ringwald, W. R. et al. Characterizing stress processes by linking big five personality states, traits, and day-to-day stressors. *J. Res. Personal.* **110**, 104487 (2024). 10.1016/j.jrp.2024.104487 PMC11067701 38708104

[42] Čeko, M., Kragel, P. A., Woo, C.-W., López-Solà, M. & Wager, T. D. Common and stimulus-type-specific brain representations of negative affect. *Nat. Neurosci.* **25**, 760–770 (2022). 35637370 10.1038/s41593-022-01082-w

[43] Han, X. et al. Effect sizes and test-retest reliability of the fMRI-based neurologic pain signature. *NeuroImage* **247**, 118844 (2022). 34942367 10.1016/j.neuroimage.2021.118844 PMC8792330

[44] Woo, C. W., Chang, L. J., Lindquist, M. A. & Wager, T. D. Building better biomarkers: brain models in translational neuroimaging. *Nat. Neurosci.* **20**, 365–377 (2017). 28230847 10.1038/nn.4478 PMC5988350

[45] Sicorello, M. et al. Affective neural signatures do not distinguish women with emotion dysregulation from healthy controls: a mega-analysis across three task-based fMRI studies. *Neuroimage Rep.* **1**, 100019 (2021). 40567870 10.1016/j.ynirp.2021.100019 PMC12172786

[46] Fried, E. I., Flake, J. K. & Robinaugh, D. J. Revisiting the theoretical and methodological foundations of depression measurement. *Nat. Rev. Psychol.* **1**, 358–368 (2022). 38107751 10.1038/s44159-022-00050-2 PMC10723193

[47] Costa, P. T. & McCrae, R. R. *Revised NEO Personality Inventory (NEO-PI-R) and NEO Five-Factor Inventory (NEO-FFI) Professional Manual* (Psychological Assessment Resources, Odessa, 1992).

[48] Cuijpers, P. et al. Economic costs of neuroticism. *Arch. Gen. Psychiatry* **67**, 1086 (2010). 20921124 10.1001/archgenpsychiatry.2010.130

[49] Ormel, J. et al. Neuroticism and common mental disorders: meaning and utility of a complex relationship. *Clin. Psychol. Rev.* **33**, 686–697 (2013). 23702592 10.1016/j.cpr.2013.04.003 PMC4382368

[50] Simonsohn, U., Simmons, J. P. & Nelson, L. D. Specification curve analysis. *Nat. Hum. Behav.* **4**, 1208–1214 (2020). 32719546 10.1038/s41562-020-0912-z

[51] Thomas Yeo, B. T. et al. The organization of the human cerebral cortex estimated by intrinsic functional connectivity. *J. Neurophysiol.* **106**, 1125–1165 (2011). 21653723 10.1152/jn.00338.2011 PMC3174820

[52] Barrett, L. F. & Satpute, A. B. Large-scale brain networks in affective and social neuroscience: towards an integrative functional architecture of the brain. *Curr. Opin. Neurobiol.* **23**, 361–372 (2013). 23352202 10.1016/j.conb.2012.12.012 PMC4119963

[53] Kragel, P. A. & LaBar, K. S. Multivariate neural biomarkers of emotional states are categorically distinct. *Soc. Cogn. Affect. Neurosci.* **10**, 1437–1448 (2015). 25813790 10.1093/scan/nsv032 PMC4631142

[54] Jong, T. de. A Bayesian Approach to the Correction for Multiplicity. Preprint at 10.31234/osf.io/s56mk (2019).

[55] Sicorello, M. et al. The elusive neural signature of emotion regulation capabilities: evidence from a large-scale consortium. 2025.08.18.670843 Preprint at 10.1101/2025.08.18.670843 (2025).

[56] Gignac, G. E. & Szodorai, E. T. Effect size guidelines for individual differences researchers. *Personal. Individ. Differ.* **102**, 74–78 (2016).

[57] Yarkoni, T., Poldrack, R. A., Nichols, T. E., Van Essen, D. C. & Wager, T. D. Large-scale automated synthesis of human functional neuroimaging data. *Nat. Methods* **8**, 665–670 (2011). 21706013 10.1038/nmeth.1635 PMC3146590

[58] Gianaros, P. J. et al. Affective brain patterns as multivariate neural correlates of cardiovascular disease risk. *Soc. Cogn. Affect. Neurosci*. 1–12 10.1093/scan/nsaa050 (2020). 10.1093/scan/nsaa050 PMC7657455 32301993

[59] Kohoutová, L. et al. Toward a unified framework for interpreting machine-learning models in neuroimaging. *Nat. Protoc.* **15**, 1399–1435 (2020). 32203486 10.1038/s41596-019-0289-5 PMC9533325

[60] Elliott, M. L. et al. What is the test-retest reliability of common task-functional MRI measures? New empirical evidence and a meta-analysis. *Psychol. Sci.* **31**, 792–806 (2020). 32489141 10.1177/0956797620916786 PMC7370246

[61] Winter, N. R. et al. A systematic evaluation of machine learning–based biomarkers for major depressive disorder. *JAMA Psychiatry* **81**, 386–395 (2024). 38198165 10.1001/jamapsychiatry.2023.5083 PMC10782379

[62] Winter, N. R. et al. Quantifying deviations of brain structure and function in major depressive disorder across neuroimaging modalities. *JAMA Psychiatry* **79**, 879–888 (2022). 35895072 10.1001/jamapsychiatry.2022.1780 PMC9330277

[63] Kragel, P. A., Reddan, M. C., LaBar, K. S. & Wager, T. D. Emotion schemas are embedded in the human visual system. *Sci. Adv.* **5**, eaaw4358 (2019). 31355334 10.1126/sciadv.aaw4358 PMC6656543

[64] Harpaintner, M., Sim, E.-J., Trumpp, N. M., Ulrich, M. & Kiefer, M. The grounding of abstract concepts in the motor and visual system: An fMRI study. *Cortex* **124**, 1–22 (2020). 31821905 10.1016/j.cortex.2019.10.014

[65] Reddan, M. C., Chang, L., Kragel, P. & Wager, T. D. Somatosensory and motor contributions to emotion representation. Preprint at 10.48550/arXiv.2411.08973 (2024).

[66] Puce, A. From motion to emotion: visual pathways and potential interconnections. *J. Cogn. Neurosci.* **36**, 2594–2617 (2024). 38527078 10.1162/jocn_a_02141 PMC11416577

[67] Koban, L., Gianaros, P. J., Kober, H. & Wager, T. D. The self in context: brain systems linking mental and physical health. *Nat. Rev. Neurosci.* **22**, 309–322 (2021). 33790441 10.1038/s41583-021-00446-8 PMC8447265

[68] Chen, X. et al. Shared but distinct functional connectome profiles underlying rumination in depressed and healthy individuals. *BMC Psychiatry* **26**, 92 (2025). 41449376 10.1186/s12888-025-07724-0 PMC12849639

[69] Robinson, O. J., Vytal, K., Cornwell, B. R. & Grillon, C. The impact of anxiety upon cognition: perspectives from human threat of shock studies. *Front. Hum. Neurosci*. **7**, 203 (2013). 10.3389/fnhum.2013.00203 PMC3656338 23730279

[70] van den Berg, J. J., Ruhé, H. G., Marquering, H. A., Reneman, L. & Caan, M. W. A. Normative amygdala fMRI response during emotional processing as a trait of depressive symptoms in the UK Biobank. *Psychol. Med.* **55**, e304 (2025). 41059632 10.1017/S0033291725101797 PMC12527494

[71] Finn, E. S. Is it time to put rest to rest? *Trends Cogn. Sci.* **25**, 1021–1032 (2021). 34625348 10.1016/j.tics.2021.09.005 PMC8585722

[72] McDermott, T. J., Kirlic, N. & Aupperle, R. L. Roadmap for optimizing the clinical utility of emotional stress paradigms in human neuroimaging research. *Neurobiol. Stress* **8**, 134–146 (2018). 29888309 10.1016/j.ynstr.2018.05.001 PMC5991342

[73] Todorov, A. The role of the amygdala in face perception and evaluation. *Motiv. Emot.* **36**, 16–26 (2012). 22448077 10.1007/s11031-011-9238-5 PMC3294209

[74] Seitz, K. I. et al. Childhood maltreatment and amygdala response to interpersonal threat in a transdiagnostic adult sample: the role of trait dissociation. *Biol. Psychiatry Cogn. Neurosci. Neuroimaging* **9**, 626–634 (2024). 38280631 10.1016/j.bpsc.2024.01.003

[75] Costa Jr, P. T. & McCrae, R. R. The revised NEO personality inventory (NEO-PI-R). in *The SAGE Handbook of Personality Theory and Assessment, Vol 2: Personality Measurement and Testing* 179–198 (Sage Publications, Inc, Thousand Oaks, CA, USA, 2008). 10.4135/9781849200479.n9.

[76] Crawford, J. R. & Henry, J. D. The positive and negative affect schedule (PANAS): construct validity, measurement properties and normative data in a large non-clinical sample. *Br. J. Clin. Psychol.* **43**, 245–265 (2004). 15333231 10.1348/0144665031752934

[77] Spielberger, C. D. *State-Trait Anxiety Inventory: Bibliography*. (Consulting Psychologists Press, Palo Alto, 1989).

[78] Beck, A. T., Steer, R. A. & Brown, G. Beck Depression Inventory–II. 10.1037/t00742-000 (1996).

[79] Gianaros, P. J. et al. An inflammatory pathway links atherosclerotic cardiovascular disease risk to neural activity evoked by the cognitive regulation of emotion. *Biol. Psychiatry* **75**, 738–745 (2014). 24267410 10.1016/j.biopsych.2013.10.012 PMC3989430

[80] Lang, P. J., Bradley, M. M. & Cuthbert, B. N. *International Affective Picture System (IAPS): Affective ratings of pictures and instruction manual.* Technical Report A-8 (University of Florida, Gainesville, FL, 2008).

[81] Ekman, P. & Friesen, W. V. *Pictures of Facial Affect*. (Consulting Psychologists Press, Palo Alto, 1976).

[82] Casey, B. J. et al. The Adolescent Brain Cognitive Development (ABCD) study: Imaging acquisition across 21 sites. *Dev. Cogn. Neurosci.* **32**, 43–54 (2018). 29567376 10.1016/j.dcn.2018.03.001 PMC5999559

[83] Miller, K. L. et al. Multimodal population brain imaging in the UK Biobank prospective epidemiological study. *Nat. Neurosci.* **19**, 1523–1536 (2016). 27643430 10.1038/nn.4393 PMC5086094

[84] Diedrichsen, J. & Shadmehr, R. Detecting and adjusting for artifacts in fMRI time series data. *NeuroImage* **27**, 624–634 (2005). 15975828 10.1016/j.neuroimage.2005.04.039 PMC1479857

[85] Geuter, S. et al. Multiple brain networks mediating stimulus–pain relationships in humans. *Cereb. Cortex* **30**, 4204–4219 (2020). 32219311 10.1093/cercor/bhaa048 PMC7264706

[86] Wager, T. D. CanlabCore. https://github.com/canlab/CanlabCore (2024).

[87] Morey, R. D. & Rouder, J. N. BayesFactor: Computation of Bayes Factors for Common Designs. *R package version* 0.9.12-4.2. (2018).

[88] Bates, S., Hastie, T. & Tibshirani, R. Cross-validation: what does it estimate and how well does it do it? *J. Am. Stat. Assoc.* **119**, 1434–1445 (2024). 39308484 10.1080/01621459.2023.2197686 PMC11412612

[89] Chen, G., Taylor, P. A. & Cox, R. W. Is the statistic value all we should care about in neuroimaging? *NeuroImage* **147**, 952–959 (2017). 27729277 10.1016/j.neuroimage.2016.09.066 PMC6591724

[90] Widaman, K. F. & Revelle, W. Thinking thrice about sum scores, and then some more about measurement and analysis. *Behav. Res. Methods* **55**, 788–806 (2023). 35469086 10.3758/s13428-022-01849-w PMC10027776

[91] Sicorello, M. et al. *The Functional Neurobiology of Dispositions Towards Negative Emotions*s. MaurizioSicorello/NeuroSquare_repo. Zenodo 10.5281/zenodo.20119671 (2026). 10.1038/s41467-026-74565-0 PMC13315715 42373635

