# Supplementary material for: The functional neurobiology of dispositions towards negative emotions
Source: Nat Commun. 2026 Jun 27;17:5622. doi: 10.1038/s41467-026-74565-0 (PMC13315715; doi:10.1038/s41467-026-74565-0)
Supplement: Supplementary file 1 — Supplementary Information [file 41467_2026_74565_MOESM1_ESM.pdf]

The functional neurobiology of dispositions towards negative emotions

-Supplements-

Content

MRI data acquisition and preprocessing.......... 2
Details on Machine Larning Algorithms.......... 2
Method for within-person prediction.......... 3
Table S1.......... 4
Table S2.......... 5
Table S3.......... 7
Table S4.......... 9
Figure S1.......... 10
Figure S2.......... 11
Figure S3.......... 12
Figure S4.......... 13
Figure S5.......... 14

MRI data acquisition and preprocessing

Functional BOLD image acquisition parameters for the facial-expression tasks were: field-of-view (FOV) = 200×200mm, matrix size = 64×64, time-to-repetition (TR) = 2000ms, time-to-echo (TE) = 29ms, and flip angle (FA) = 90°. Thirty-four slices per volume were collected along an inferior-to-superior encoding direction, with each volume having a 3mm thickness and no gap. A total of 195 and 273 BOLD signal volumes were collected throughout the facial-expression tasks using PFA and NIM-STIM images, respectively. Functional BOLD image acquisition parameters for the scenes task were: FOV = 205×205mm, matrix size = 64×64, TR = 2000ms, TE = 28ms, and FA = 90°. The entire task duration was 11 min and 16 sec (15 'Look neutral' trials; 15 'Look negative' trials; 15 'Decrease negative' trials). Images were presented such that no more than 2 identical trial types ("Look negative" or "Decrease negative") were consecutive, and no more than 4 unpleasant images were consecutive. Thirtynine slices per volume were collected along an inferior-to-superior encoding direction, with each volume having a 3mm thickness and no gap (338 task volumes in total). A 6-sec countdown preceded task onset. The 3 volumes of this countdown were not modeled in the design matrix, nor were remaining 3 volumes collected after the offset of the final rest period (344 functional run volumes in total). To co-register BOLD signal volumes, T1-weighted magnetization prepared rapid gradient echo (MPRAGE) images were acquired over 7 min and 17 sec by these parameters: FOV = 256×208mm, matrix size = 256×208, TR = 2100ms, inversion time (TI) = 1100ms, TE = 3.31ms, and FA = 8° (192 slices, 1mm thickness, no gap).

All fMRI data for the facial-expression and scenes tasks were preprocessed with statistical parametric mapping software (SPM12; http://www.fil.ion.ucl.ac.uk/spm). For spatial preprocessing, T1-weighted MPRAGE images were classified into 6 tissue types. Bias-corrected and deformation field maps were then computed. Functional images were realigned to the first image of the series by 6-parameter rigid-body transformation, using the re-slice step to match the first image on a voxel-by-voxel basis. Before realignment, slice-timing correction was applied to scenes fMRI task data to account for acquisition time variation in this event-related design. Realigned images were co-registered to each participant's skull-stripped and biased-corrected MPRAGE image. Co-registered images were then normalized to Montreal Neurological Institute (MNI) space. Normalized images were then smoothed by a 6mm fullwidth-at-half-maximum (FWHM) Gaussian kernel.

Details on Machine Larning Algorithms

Model performance in the training sample was estimated using 2x5x5 nested cross-validation the inner folds being used for hyperparameter optimization, and the outer folds for independent testing using the chosen optimal hyperparameter (PLS/PCR: number of components between one and the sample size of the innermost training fold optimized using Bayesian parameter optimization, SVM: Slack parameter C optimized using grid search, RF: mtry with grid search between one and total number of variables after dimensionality reduction using PCA with the number of components equal to the sample size of the innermost training fold). The procedure was repeated two times for more stability. Product-

moment correlations were used as a performance metric, as the aim was to train a pattern for scale-independent correlates of negative affectivity, which can be used across different studies with different scanning parameters or different questionnaires. The resulting 10 product-moment correlations (two repeats times five outer folds) were Fisher-transformed, averaged, and the average back-transformed for the final model estimate. The model configuration with the highest average correlation was chosen for evaluation in the hold-out sample. Before evaluation, the model was retrained on the full training data using an optimal hyperparameter chosen based on 5-fold cross-validation (corresponding to the inner folds of the nested procedure). Using this final model, neuroticism scores of the hold-out sample were correlated with model predictions, generated by taking the dot product between machine learning pattern and fMRI data. This final correlation was supplemented with p-values and Bayes factors, both one-sided.

Method for within-person prediction

Within-person prediction had been previously performed on the same dataset for the creation of the PINES signature by Chang and colleagues (2015; see main manuscript). They prepared one beta image for each person and rating to a negative picture, i.e., as ratings varied between 1 and 5, each participant is associated with five beta images. These images are openly available on neurovault: https://neurovault.org/collections/1964/. In accordance with the previous study, we stacked the images on top of each other in a single data matrix. Then, we performed 10-fold cross-validation, leaving out 10% of participants in each iteration to avoid dependencies in training and test data. Nine folds were used for training a predictive model based on the specifications of the best model we identified for individual differences, i.e., support vector regression after image-wise centering. In the remaining test fold, a correlation between actual and predicted ratings was calculated separately for each participant. This procedure was repeated until each participant was part of a test fold, providing person-specific correlations for the whole sample. We report the mean value and standard deviation for these person-wise correlations in the main paper.

Table S1

Theory-driven neural associations with neuroticism

	SCENES (N = 332)			FACES (N = 424)		
	r	95% CI	BF_{01}	r	95% CI	BF_{01}
			Regions			
Averaged signal						
Amygdala-L	.03	[-.08, .14]	19.4	.07	[-.03, .16]	10.5
Amygdala-R	.05	[-.06, .15]	16.4	.06	[-.04, .15]	12.6
aInsula-L	-.05	[-.15, .06]	16.1	.03	[-.06, .13]	20.4
aInsula-R	-.06	[-.17, .05]	12.7	.05	[-.05, .14]	16.6
dACC-L	-.11	[-.22, .00]	3.4	.04	[-.06, .13]	18.7
dACC-R	-.11	[-.22, -.00]	2.84	.04	[-.05, .13]	17.0
PLS Models[a]						
Amygdala	-.05	[-.24, .15]	11.5	.13	[-.06, .32]	5.3
aInsula	-.05	[-.24, .15]	11.4	.02	[-.18, .21]	12.6
dACC	.04	[-.16, .23]	11.9	.09	[-.11, .28]	8.6
Best region						
Cerebellum/ Diencephalon	-.12	[-.26, .03]	4.95	.07	[-.06, .21]	9.5
			Networks			
Averaged Signal						
Visual	-.02	[-.13, .09]	21.6	.04	[-.06, .13]	19.4
Somatomotor	-.02	[-.12, .09]	21.9	.11	[.02, .20]	1.9
dAttention	-.03	[-.13, .08]	20.5	.07	[-.03, .16]	9.5
vAttention	-.04	[-.15, .07]	17.5	.07	[-.03, .16]	9.9
Limbic	-.01	[-.12, .09]	22.2	.12	[.03, .22]	1.0
Frontoparietal	-.10	[-.20, .01]	4.7	.04	[-.05, .14]	17.0
Default-Mode	-.07	[-.18, .04]	10.1	.06	[-.04, .15]	12.5
Linear Regression						
All networks[b]	-.03	.467	>100	.12	.071	>100
Random Forest						
All networks[b]	.10	.059		-.06	.710	
			Signatures			
PINES	-.09	[-.20, 0.02]	5.5	.07	[-.03, .16]	9.5
VIFS	.04	[-.07, .15]	17.6	-.03	[-.13, .07]	21.3
Fear	.05	[-.06, .15]	16.2	.01	[-.09, .10]	25.5
Anger	.01	[-.10, .12]	22.4	.05	[-.05, .14]	16.1
Sadness	-.03	[-.13, .08]	20.6	.05	[-.05, .14]	15.4

Note. BF_{01} = Bayes factor of the null hypothesis over the alternative hypothesis. Bayes factors were calculated with the bayesFactor toolbox in matlab.

[a]The proportion of in-region voxels weights with positive signs ranged between 49-51%.

[b]For the linear multiple regression approach, the multiple correlation coefficient is reported, calculated as the square-root of adjusted R^2. Here, p-values are reported instead of confidence intervals, as confidence intervals for (unadjusted) R^2 cannot contain zero and are therefore harder to interpret in terms of statistical significance. Similarly, p-values are reported for the random forest approach, as it was preregistered to be conducted on the whole sample with out-of-bag prediction for which (to our knowledge) currently are no valid confidence intervals available. The p-value is for a one-sided test, as negative correlations between predictions and actual values are not meaningful.

Table S2

Clusters of voxels with positive weights at α = .05 (uncorrected) with cluster threshold 50

Region	Volume [mm³]	MNI			Z_{max}-Statistic	% covered
		X	Y	Z		
Basal ganglia	1616	-14	10	10	3.85	70
Brainstem	1472	-2	-26	-10	3.98	23
Cerebellum	2440	12	-42	-18	4.03	62
Cerebellum	4264	0	-42	-52	3.73	25
Cerebellum	5488	30	-68	-20	4.18	44
Cerebellum	6944	18	-68	-38	3.97	31
Cerebellum	7472	-24	-54	-22	3.44	26
Cerebellum	7776	-22	-70	-38	4.19	43
Cortex Default ModeA	1064	-8	62	8	3.95	85
Cortex Default ModeA	2984	-24	24	50	3.61	35
Cortex Default ModeB	1288	16	52	32	3.63	54
Cortex Default ModeB	1424	-10	18	56	3.20	41
Cortex Default ModeB	1648	-54	-8	-12	3.34	45
Cortex Default ModeC	1296	-36	-28	-16	3.79	38
Cortex Default ModeC	2208	36	-82	12	2.77	31
Cortex Dorsal AttentionA	1472	-44	-36	-28	2.70	35
Cortex Dorsal AttentionA	1704	14	-56	62	3.93	31
Cortex Dorsal AttentionB	1592	-32	-52	66	4.33	50
Cortex Dorsal AttentionB	1608	66	-40	4	3.13	19
Cortex Fronto ParietalA	2016	46	36	18	4.14	41
Cortex Fronto ParietalB	1560	-58	-48	-14	3.94	85
Cortex Fronto ParietalC	9072	8	-76	40	4.25	11
Cortex Limbic	1312	-24	2	-24	3.09	38
Cortex Limbic	1616	34	-26	-26	3.57	45
Cortex Limbic	2360	26	-4	-42	4.37	42
Cortex Limbic	2888	-28	0	-42	4.09	51
Cortex Limbic	2984	18	58	-16	3.89	47
Cortex Limbic	4808	46	14	-30	5.04	66
Cortex Limbic	7456	54	-18	-30	4.26	40
Cortex Limbic	7656	-28	40	-14	3.65	10
Cortex SomatomotorB	2112	52	10	26	3.28	47
Cortex Temporal Parietal	1064	-48	6	-6	4.16	33
Cortex Temporal Parietal	1984	52	-50	20	3.28	29
Cortex Temporal Parietal	3096	-50	-34	10	3.20	18
Cortex Ventral AttentionA	2480	54	2	0	3.32	22
Cortex Ventral AttentionA	4184	40	-14	-2	4.39	18
Cortex Ventral AttentionB	2024	0	6	30	2.95	9
Cortex Ventral AttentionB	2232	0	34	14	4.04	30
Cortex Visual Central	1416	20	-86	-12	2.87	42
Cortex Visual Central	2264	2	-84	22	3.91	26

Cortex Visual Peripheral	1336	26	-62	6	4.04	51
Cortex Visual Peripheral	1904	-4	-76	10	3.45	55
Cortex Visual Peripheral	2336	-26	-96	2	3.33	35
Cortex Visual Peripheral	2840	6	-60	0	4.28	31

Note. The “% covered” column indicates the percent of voxels in a cluster cover by a specific region label. Automated labelling was performed with the canlab toolbox using the region() and table() functions.

Table S3

Clusters of voxels with negative weights at α = .05 (uncorrected) with cluster threshold 50

Region	Volume [mm³]	MNI X	MNI Y	MNI Z	Z_{max}-Statistic	% covered
Basal ganglia	1328	12	22	2	-4.00	39
Basal ganglia	1504	-22	12	-4	-3.81	67
Basal ganglia	5272	6	4	-8	-3.81	10
Cerebellum	1024	38	-72	-50	-2.90	95
Cerebellum	1024	36	-52	-48	-2.85	45
Cerebellum	1904	-30	-38	-30	-3.18	63
Cerebellum	2136	8	-58	-48	-4.21	55
Cerebellum	2520	-18	-88	-20	-3.41	43
Cortex Default ModeA	2128	-6	38	14	-3.44	24
Cortex Default ModeA	4008	-34	-72	42	-3.52	31
Cortex Default ModeA	4224	0	48	-8	-3.89	26
Cortex Default ModeB	1056	50	18	0	-3.41	52
Cortex Default ModeB	1472	-60	-16	-4	-3.86	29
Cortex Default ModeB	1696	-48	28	-2	-4.06	60
Cortex Default ModeB	12192	-40	-14	48	-4.14	10
Cortex Default ModeC	2304	8	-44	18	-3.36	22
Cortex Dorsal AttentionA	10240	46	-70	0	-4.44	17
Cortex Dorsal AttentionB	1456	14	-44	70	-4.20	54
Cortex Dorsal AttentionB	16896	44	18	46	-4.86	10
Cortex Fronto ParietalA	1360	-10	-6	68	-2.97	40
Cortex Fronto ParietalB	1136	-50	-54	28	-2.98	42
Cortex Fronto ParietalB	1256	54	-44	46	-3.39	57
Cortex Fronto ParietalC	1464	-12	-58	22	-3.15	26
Cortex Fronto ParietalC	5184	2	-48	38	-4.02	21
Cortex Limbic	1528	40	-16	-22	-3.17	28
Cortex Limbic	1760	10	36	-22	-4.17	54
Cortex Limbic	3168	34	14	-22	-4.45	29
Cortex SomatomotorA	1272	60	-10	38	-3.08	55
Cortex SomatomotorB	2208	50	-32	16	-4.00	25
Cortex SomatomotorB	2224	-64	-14	20	-3.55	29
Cortex SomatomotorB	2232	-44	-14	14	-3.46	39
Cortex Ventral AttentionA	1080	-42	-4	-2	-3.00	67
Cortex Ventral AttentionA	1400	-38	16	-2	-3.06	61
Cortex Ventral AttentionA	10528	-2	18	44	-4.73	17
Cortex Ventral AttentionB	1280	-18	46	-18	-3.57	40
Cortex Ventral AttentionB	1312	40	46	4	-2.84	46

Cortex Ventral AttentionB	1464	36	22	10	-3.59	43
Cortex Visual Central	1264	16	-96	16	-3.25	43
Cortex Visual Central	1312	40	-44	-22	-3.10	34
Cortex Visual Central	1472	-44	-76	4	-4.42	40
Cortex Visual Central	3552	2	-68	-4	-4.47	27
Cortex Visual Peripheral	2312	12	-98	2	-3.21	71
Cortex Visual Peripheral	3200	-4	-88	-10	-3.87	52
Diencephalon	5768	14	-32	6	-4.49	15

Note. The "% covered" column indicates the percent of voxels in a cluster cover by a specific region label. Automated labelling was performed with the canlab toolbox using the region() and table() functions.

Table S4

Correlations between stress vulnerability and literature-based neural measures

		Uncentered				Centered			
Category	Measure	*r*	95% *CI*	*p*	BF_{01}	*r*	95% *CI*	*p*	BF_{01}
Regions	Amygdala	0.03	[-0.07, 0.14]	0.552	19.3	0.12	[0.01, 0.22]	0.031	2.25
	dACC	-0.16	[-0.26, -0.06]	0.003	0.28	-0.17	[-0.27, -0.07]	0.001	0.15
	aInsula	-0.07	[-0.18, 0.03]	0.181	9.43	-0.02	[-0.13, 0.08]	0.648	20.8
Networks	Visual	-0.06	[-0.17, 0.04]	0.243	11.7	-0.00	[-0.11, 0.11]	0.991	23.1
	Somatomotor	-0.02	[-0.13, 0.08]	0.677	21.2	0.06	[-0.05, 0.16]	0.282	12.9
	dAttention	-0.12	[-0.23, -0.02]	0.022	1.67	-0.11	[-0.22, -0.01]	0.037	2.62
	vAttention	-0.06	[-0.16, 0.05]	0.312	13.9	0.02	[-0.09, 0.12]	0.756	22.0
	Limbic	-0.05	[-0.15, 0.06]	0.377	15.6	0.05	[-0.06, 0.16]	0.370	15.4
	Frontoparietal	-0.16	[-0.26, -0.05]	0.004	0.34	-0.19	[-0.29, -0.08]	<0.001	0.06
	Default	-0.13	[-0.23, -0.02]	0.016	1.28	-0.14	[-0.24, -0.03]	0.011	0.89
Signatures	PINES	-0.08	[-0.19, 0.02]	0.126	7.19	-0.08	[-0.18, 0.03]	0.158	8.52
	VIFS	0.02	[-0.09, 0.12]	0.761	22.0	-0.00	[-0.11, 0.10]	0.934	23.0
	Fear	-0.11	[-0.22, -0.01]	0.038	2.71	-0.09	[-0.20, 0.01]	0.087	5.36
	Anger	-0.03	[-0.14, 0.08]	0.570	19.6	0.00	[-0.11, 0.11]	0.975	23.1
	Sadness	0.15	[0.04, 0.25]	0.006	0.53	0.14	[0.04, 0.25]	0.008	0.70

Note. *N*=338. All *p*-values and Bayes factors are two-sided and uncorrected. aInsula = anteriore Insula. v/dAttention = ventral/dorsal Attention Network. PINES = Picture Induced Negative Emotion Signature, VIFS = Visually Induced Fear Signature.

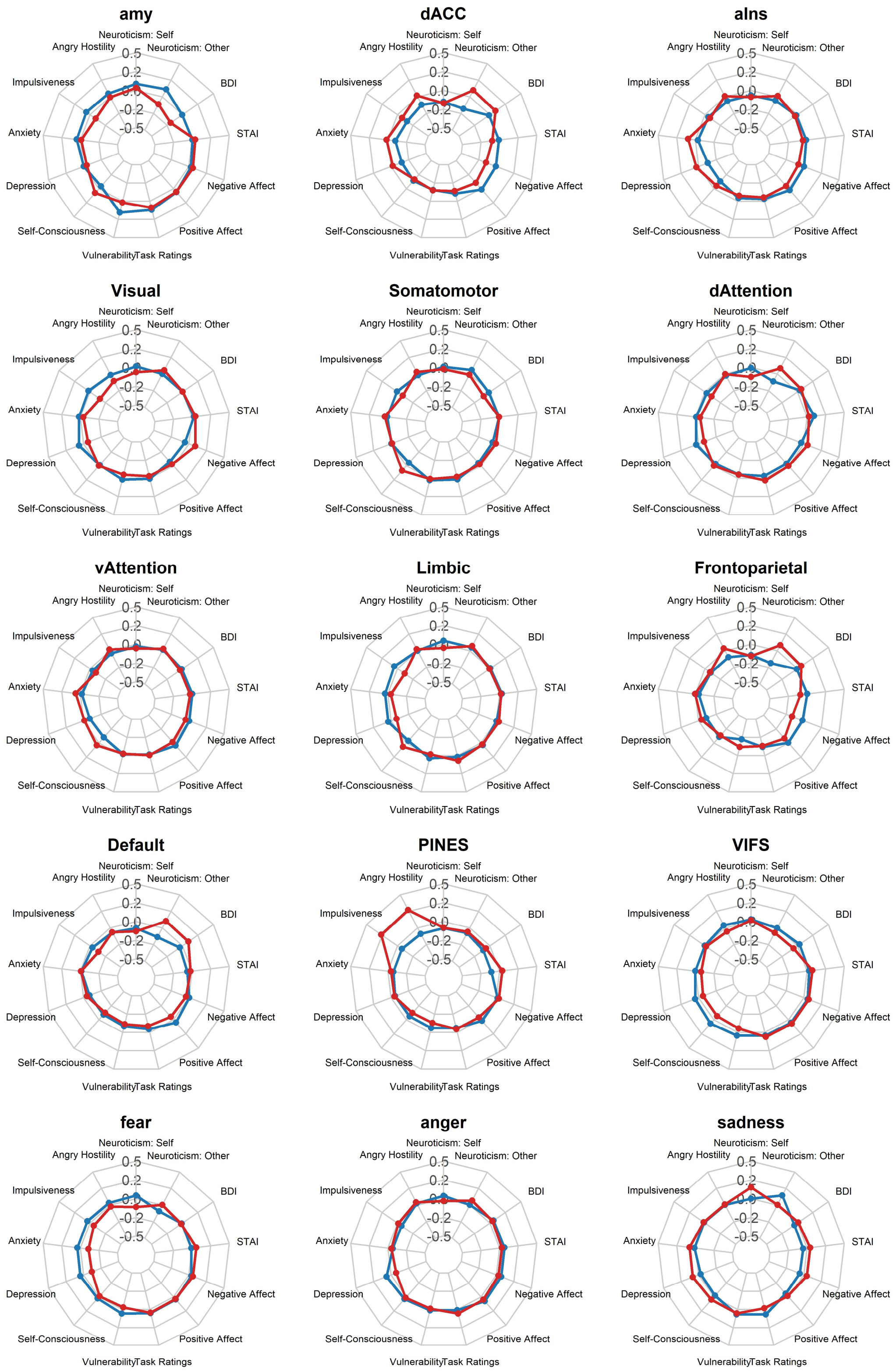

Figure S1. Correlations between neural measures and psychological constructs for scenes. Correlations between responses of neural measures (titles) and constructs (edges) in the scenes task (negative versus neutral trials) separately for centered (blue lines) and uncentered (red lines) images. Given the sample size, correlations above r = .11 are statistically significant (two-sided; uncorrected). For further descriptions of constructs, neural measures, and abbreviations, see the figures in the main text.

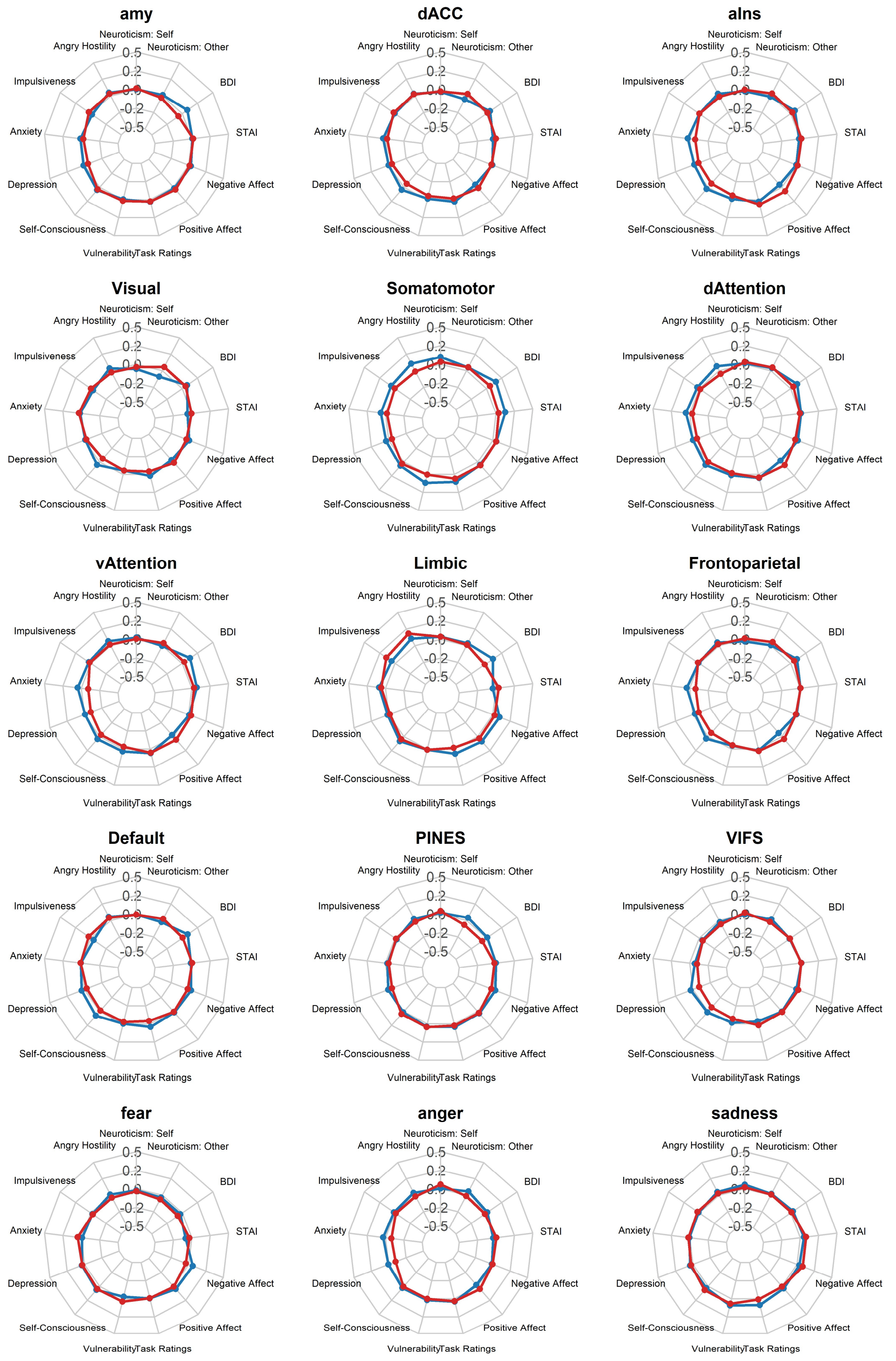

Figure S2. Correlations between neural measures and psychological constructs for faces. Correlations between responses of neural measures (titles) and constructs (edges) in the faces task (negative faces versus shapes) separately for centered (blue lines) and uncentered (red lines) images. Given the sample size, correlations above r = .10 are statistically significant (two-sided; uncorrected). For further descriptions of constructs, neural measures, and abbreviations, see the figures in the main text.

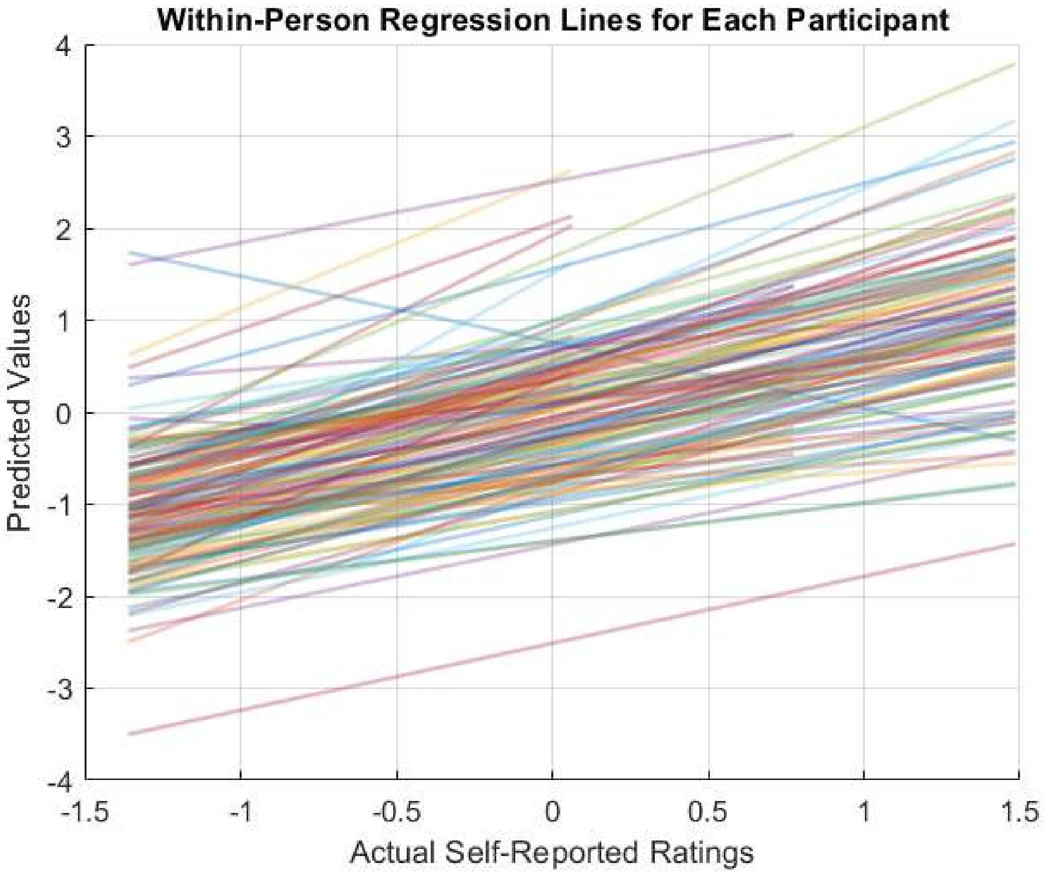

Figure S3. Within-person decoding of task-based affective ratings.
Within-person regression lines for the association between actual state-based self-report rating of negative affect and predicted values from a support vector regression.

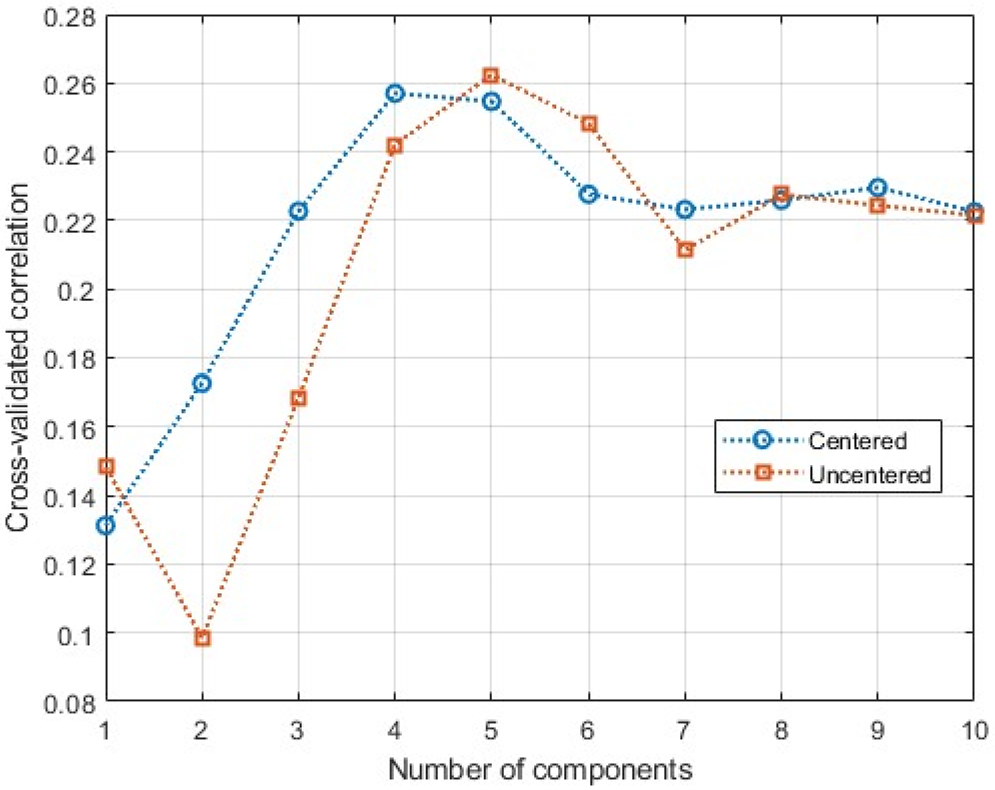

Figure S4. Number of components needed for prediction.

Task-based affective self-reports during the scenes task were predicted from partial least squares (PLS) models with an increasing number of components. This figure shows the cross-validated correlations between predicted and actual responses as a function of the number of components in centered and uncentered contrast images, respectively. Centering refers to subtracting the average whole-brain response of each participant from all voxels in their respective contrast image. This plot shows that PLS models on uncentered data perform equally well as on centered data after 4 components. Still, the model on centered data needs less components to reach a performance plateau. This pattern fits the hypothesis that PLS on uncentered data first has to remove confounding by whole-brain responses before "catching up" to the PLS model on centered data, where these nuisance signals are already removed. Further evidence for this explanation is provided in Figure S5.

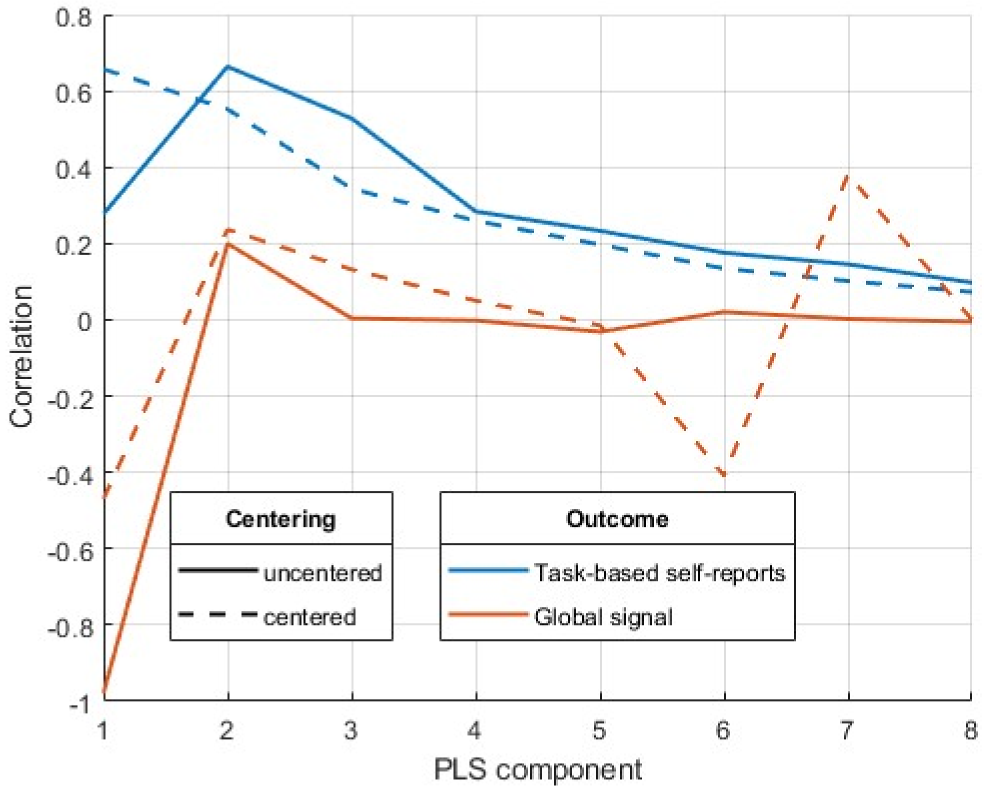

Figure S5. Interpretation of predictive components.

Correlations (y-axis) between participants' component scores on the first eight PLS components (x-axis) and either task-based self-reports (blue) or global signal responses (red). For the PLS model on centered data, the correlation between the component scores and task-based self-reports is monotonically decreasing (dashed blue line). This is consistent with the idea that this model can extract useful information from the data already starting with the first component. For uncentered data, on the other hand, the first component has a relatively low correlation with the predicted task-based reports, rising afterwards, indicating the first component is largely capturing noise (solid blue line). This is further supported by correlations between PLS component scores and global signals responses. The first component of the uncentered model is almost perfectly negatively correlated with global signal responses. Hence, overall, these patterns of Figure S4 and S5 confirm that PLS on uncentered data is implicitly removing global signal responses and therefore performs similarly to PLS on centered data, although it might need slightly more components.

Three additional observations might be relevant for this pattern. Firstly, Figures S4 and S5 both indicated that the first PLS component of the uncentered model already had some predictive value. Follow-up analyses showed that this is the case because the task-based affective responses are correlated with the global signal responses at $r = -.14$. Hence, people who responded more strongly to stimuli based on self-reports actually had *smaller* whole-brain responses. This speaks against the explanation that whole-brain responses might capture task-related hyperarousal to stimulation. Secondly, for the uncentered model, only the first two components were related to global signal. For the following components, it is perfectly removed (solid red line). In contrast, for the centered model, later components were again related to global signals, indicating that implicit PLS denoising might lead to cleaner solutions than manual denoising by image centering. Thirdly, these implicit mechanisms should generalize to PCR and SVR, but not random forest regression, as they all share an implicit similarity to L2 regularization.
