# Supplementary material for: The functional neurobiology of dispositions towards negative emotions
Source: Nat Commun. 2026 Jun 27;17:5622. doi: 10.1038/s41467-026-74565-0 (PMC13315715; doi:10.1038/s41467-026-74565-0)
Supplement: Supplementary file 2 — Reporting Summary [file 41467_2026_74565_MOESM2_ESM.pdf]

nature portfolio

Corresponding author(s): M. Sicorello, Tor D. Wager

Last updated by author(s): May 11, 2026

Reporting Summary

Nature Portfolio wishes to improve the reproducibility of the work that we publish. This form provides structure for consistency and transparency in reporting. For further information on Nature Portfolio policies, see our Editorial Policies and the Editorial Policy Checklist.

Statistics

For all statistical analyses, confirm that the following items are present in the figure legend, table legend, main text, or Methods section.

n/a	Confirmed	
☐	☒	The exact sample size (*n*) for each experimental group/condition, given as a discrete number and unit of measurement
☐	☒	A statement on whether measurements were taken from distinct samples or whether the same sample was measured repeatedly
☐	☒	The statistical test(s) used AND whether they are one- or two-sided<br>*Only common tests should be described solely by name; describe more complex techniques in the Methods section.*
☐	☒	A description of all covariates tested
☐	☒	A description of any assumptions or corrections, such as tests of normality and adjustment for multiple comparisons
☐	☒	A full description of the statistical parameters including central tendency (e.g. means) or other basic estimates (e.g. regression coefficient) AND variation (e.g. standard deviation) or associated estimates of uncertainty (e.g. confidence intervals)
☐	☒	For null hypothesis testing, the test statistic (e.g. *F*, *t*, *r*) with confidence intervals, effect sizes, degrees of freedom and *P* value noted<br>*Give P values as exact values whenever suitable.*
☐	☒	For Bayesian analysis, information on the choice of priors and Markov chain Monte Carlo settings
☒	☐	For hierarchical and complex designs, identification of the appropriate level for tests and full reporting of outcomes
☐	☒	Estimates of effect sizes (e.g. Cohen's *d*, Pearson's *r*), indicating how they were calculated

Our web collection on statistics for biologists contains articles on many of the points above.

Software and code

Policy information about availability of computer code

Data collection	E-Prime software (Psychology Software Tools, Sharpsburg, PA) was used to present stimuli and record behavioral responses in the facial expression and IAPS tasks
Data analysis	Matlab 2023b, R v4.2.3, Canlab Coretools (2024)

For manuscripts utilizing custom algorithms or software that are central to the research but not yet described in published literature, software must be made available to editors and reviewers. We strongly encourage code deposition in a community repository (e.g. GitHub). See the Nature Portfolio guidelines for submitting code & software for further information.

Data

Policy information about availability of data

All manuscripts must include a data availability statement. This statement should provide the following information, where applicable:

- Accession codes, unique identifiers, or web links for publicly available datasets
- A description of any restrictions on data availability
- For clinical datasets or third party data, please ensure that the statement adheres to our policy

Deidentified psychological assessment data and neuroimaging data can be accessed, respectively, via https://github.com/MaurizioSicorello/NeuroSquare_repo and https://identifiers.org/neurovault.collection:5802

Research involving human participants, their data, or biological material

Policy information about studies with human participants or human data. See also policy information about sex, gender (identity/presentation), and sexual orientation and race, ethnicity and racism.

Reporting on sex and gender	Self-assigned gender was reported in the manuscript, with only two options. In the faces task, 52.1% of participants were women. In the scenes task, 48.8% were women. Only aggregate analyses over the whole sample were performed, given sample size considerations and as there is no convincing indication that major personality traits should be differently represented in the brains of men vs women.
Reporting on race, ethnicity, or other socially relevant groupings	A coarse assessment of self-assigned race was performed to test the representativeness of the sample. the majority endorsed white/caucasian (about 80%), followed by african-american (about 18%).
Population characteristics	Additionally to the demographic information given above, the average age was 42.8 years in the faces task (SD = 7.4) and 41.3 in the scenes task (SD = 7.1). The sample was a community sample without specific restrictions concerning diagnosis.
Recruitment	Participants of AHAB-2 and PIP were recruited by mass-mailings to residents of Western Pennsylvania (principally Allegheny County) identified from motor vehicle, voting registration, and other public domain lists between 2008 - 2011 (AHAB-2) and 2008 - 2014 (PIP). The collective period of data collection extended from 2008 to 2014. All participants provided informed consent. In both PIP and AHAB-II, potential self-selection bias may have arisen because participants were community volunteers willing to undergo neuroimaging and psychophysiological testing; however, the demographic composition was broadly representative, and variance in the main outcome, neuroticism, was slightly above representative norms, suggesting that any such bias is unlikely to have restricted trait variability or selectively inflated brain–trait associations.
Ethics oversight	University of Pittsburgh Human Research Protection Office

Note that full information on the approval of the study protocol must also be provided in the manuscript.

Field-specific reporting

Please select the one below that is the best fit for your research. If you are not sure, read the appropriate sections before making your selection.

[x] Life sciences [ ] Behavioural & social sciences [ ] Ecological, evolutionary & environmental sciences

For a reference copy of the document with all sections, see nature.com/documents/nr-reporting-summary-flat.pdf

Life sciences study design

All studies must disclose on these points even when the disclosure is negative.

Sample size	The faces task had a sample size of N = 424; the scenes task had a sample size of N = 338. The papers reports secondary analyses on an existing dataset with fixed sample size. Sample sizes of the hold-out samples were determined with gpower to have statistical power of 90% to detect a true correlation between pattern and neuroticism of r ≈ .30 in a one-tailed test.
Data exclusions	One participant in the faces task was a multivariate outlier according to Bonferroni-Holm-corrected Mahalanobis distance and therefore excluded from analyses on that task.
Replication	Machine learning analyses were successfully replicated on unseen stratified hold-out data split from the original data.
Randomization	N/A
Blinding	N/A

Reporting for specific materials, systems and methods

We require information from authors about some types of materials, experimental systems and methods used in many studies. Here, indicate whether each material, system or method listed is relevant to your study. If you are not sure if a list item applies to your research, read the appropriate section before selecting a response.

Materials & experimental systems

n/a	Involved in the study
☒	☐ Antibodies
☒	☐ Eukaryotic cell lines
☒	☐ Palaeontology and archaeology
☒	☐ Animals and other organisms
☒	☐ Clinical data
☒	☐ Dual use research of concern
☒	☐ Plants

Methods

n/a	Involved in the study
☒	☐ ChIP-seq
☒	☐ Flow cytometry
☐	☒ MRI-based neuroimaging

Plants

Seed stocks

Report on the source of all seed stocks or other plant material used. If applicable, state the seed stock centre and catalogue number. If plant specimens were collected from the field, describe the collection location, date and sampling procedures.

Novel plant genotypes

Describe the methods by which all novel plant genotypes were produced. This includes those generated by transgenic approaches, gene editing, chemical/radiation-based mutagenesis and hybridization. For transgenic lines, describe the transformation method, the number of independent lines analyzed and the generation upon which experiments were performed. For gene-edited lines, describe the editor used, the endogenous sequence targeted for editing, the targeting guide RNA sequence (if applicable) and how the editor was applied.

Authentication

Describe any authentication procedures for each seed stock used or novel genotype generated. Describe any experiments used to assess the effect of a mutation and, where applicable, how potential secondary effects (e.g. second site T-DNA insertions, mosiacism, off-target gene editing) were examined.

Magnetic resonance imaging

Experimental design

Design type

task-based event related and block design

Design specifications

Scenes task: Participants saw 30 unpleasant and 15 neutral IAPS images. Trials were comprised of a 2s cue ('Look' or 'Decrease'), followed by a 7s IAPS. After image viewing, participants rated their emotional state ('How negative do you feel?') on a 5-point Likert-type scale in a 4s rating period (1 = neutral, 5 = strongly negative). A variable (1–3s) rest period followed each rating period. The entire task duration was 11 min and 16s (15 'Look neutral' trials; 15 'Look negative' trials; 15 'Decrease negative' trials).
Faces task: Participants completed four blocks of a facial expression-matching-to sample condition, which was interleaved with five blocks of a shape-matching (sensorimotor) control condition. Each block consisted of six trials (three fear, three anger; three all-male, three all-female), and each trial lasted for 4s (1.5, 3.5 and 5.5s variable inter-trial interval; ITI). Each trio of shapes was shown for 4s with a 2s ITI. The total task length was 6 min and 36 seconds.

Behavioral performance measures

In the scenes task, after image viewing, participants rated their emotional state ('How negative do you feel?') on a 5-point Likert-type scale in a 4s rating period (1 = neutral, 5 = strongly negative). Face matching in the faces task only served as an aid to focus on the task and was not further analyzed.

Acquisition

Imaging type(s)

functional

Field strength

3T

Sequence & imaging parameters

Functional BOLD image acquisition parameters for the facial-expression tasks were: field-of-view (FOV) = 200×200mm, matrix size = 64×64, time-to-repetition (TR) = 2000ms, time-to-echo (TE) = 29ms, and flip angle (FA) = 90°. Thirty-four slices per volume were collected along an inferior-to-superior encoding direction, with each volume having a 3mm thickness and no gap. A total of 195 and 273 BOLD signal volumes were collected throughout the facial-expression tasks using PFA and NIM-STIM images, respectively.
Functional BOLD image acquisition parameters for the IAPS task were: FOV = 205×205mm, matrix size = 64×64, TR = 2000ms, TE = 28ms, and FA = 90°.

Area of acquisition

whole-brain

Diffusion MRI ☐ Used ☒ Not used

Preprocessing

Preprocessing software	SPM12
Normalization	DARTEL
Normalization template	MNI305
Noise and artifact removal	Each GLM additionally<br>The first-level model included six motion regressors of no interest and a high-pass temporal filter (128s) to correct for low frequency drift.
Volume censoring	N/A

Statistical modeling & inference

Model type and settings	Univariate general linear models (GLMs) were estimated to compute condition or event contrast maps that were later used for multivariate prediction analysis.
Effect(s) tested	In both tasks, beta images of the negative emotional condition were used (scenes or faces, respectively). For the main analysis, the neutral control pictures were subtracted from these images. These were used for multivariate prediction analyses.

Specify type of analysis: ☐ Whole brain ☐ ROI-based ☒ Both

Anatomical location(s): Regions were preregistered and based on the CanlabCore (2024) atlas.

Statistic type for inference<br>(See Eklund et al. 2016)	For characterization of the multivariate patterns, first, voxel-wise analyses were performed, follwed by cluster-level analyses: "No voxel-wise regression weights of the vulnerability-pattern passed FDR correction at q = .05. To still give some intuition about the location of the most strongly contributing voxels, we applied a more lenient thresholding procedure used in a previous study on this dataset at α = .05 and a minimum cluster-size of 50 contiguous voxels."
Correction	FDR and cluster-based correction

Models & analysis

n/a	Involved in the study
☒	☐ Functional and/or effective connectivity
☒	☐ Graph analysis
☐	☒ Multivariate modeling or predictive analysis

Multivariate modeling and predictive analysis	Voxel-wise beta estimates served as independent variables, questionnaire-based personality traits as dependent (criterion) variables. Feature selection was mostly performed implicitly by the algorithms (e.g., PLS, PCR) based on hyperparameter tuning of latent components using nested cross-validation. PCA was used for dimension reduction for random forest algorithms. Correlations were used a performance measure for cross-validation and analyses on the hold-out set, as the goal was to find a direction-sensitive biomarker-like correlate of negative emotional traits, which is not dependent on exact prediction, as fMRI parameters will differ between studies and affect the scale of predicted values.
