# Supplementary material for: The functional neurobiology of dispositions towards negative emotions
Source: Nat Commun. 2026 Jun 27;17:5622. doi: 10.1038/s41467-026-74565-0 (PMC13315715; doi:10.1038/s41467-026-74565-0)
Supplement: Supplementary file 3 — Transparent Peer Review file [file 41467_2026_74565_MOESM3_ESM.pdf]

nature portfolio

Peer Review File

The functional neurobiology of dispositions towards negative emotions

Corresponding Author: Dr Maurizio Sicorello

This file contains all reviewer reports in order by version, followed by all author rebuttals in order by version.

Version 0:

Reviewer comments:

Reviewer #1

(Remarks to the Author)
Sicorello et al. present a manuscript on the prediction (in machine-learning terms) of affective traits from neural (fMRI) data. This topic is important because it addresses the neural correlates of negative affect traits from both basic and applied perspectives. Moreover, the authors employ sophisticated and up-to-date analytical methods. Overall, the manuscript has potential, and the results (even if largely null or modest) are of interest. However, the manuscript requires substantial editing and revision before it can be considered for publication.

These are some points that the author may take in consideration. I hope this is helpful.

1. Clarity and conceptual precision
I found the manuscript overall difficult to follow. The abstract and introduction are confusing, and the use of several terms and concepts is imprecise. For example, the authors appear to use “depressivity” and “depression” interchangeably, and the term “prediction” is inconsistently applied. In addition, after reading the introduction, it remains unclear how the authors distinguish between trait and state constructs.

2. Study goals
The exact goals of the study are not clearly stated, which may partly stem from the writing issues noted above. The manuscript should explicitly define its aims and hypotheses.

3. Writing and style
The text needs thorough editing for grammar, syntax, and word choice (there are several typos, missing words, and grammatical errors). Some expressions are incorrect or confusing. For instance, in the sentence “In contrast to single regions and resting-state networks, affective neural signatures…”, the intended comparison is unclear, because the authors are not referring to regions per se but to measures (of brain activation). Similarly, the phrase “for neural salience regions based on structure, functional connectivity, and responsivity to negative stimuli” conflates a technique (functional connectivity) with a process (responsivity).

4. Presentation of results and figures
The presentation of the results—including the figures—needs major improvement.
Figure 1: It is unclear that fMRI data are used; “regions” is ambiguous (resting-state or task-based?), and the sample is not described. The meaning of “added choices” and “Six sub-facets” is also unclear.
Figure 2: The acronyms “PINES” and “VIFS” are never defined.
Figure 3: The reader first encounters “virtual lesion” results without any prior explanation of the term. Some results (“Other self-report” and “combined.”) also are new for the reader.

5. Preregistration
A key strength of the study would be preregistration of its aims and hypotheses. However, I could not see this information in the link provided by the authors in page 5 (In fact, I could not see any preregistration document)

6. Sample description
The authors state in the abstract that the results are based on “a large community sample with population-representative

variance in the targeted traits." Yet, neither the Participants section nor Table 1 clearly supports this claim. In particular, descriptive statistics for the questionnaire scores are missing from the main text.

7. Specific issues requiring clarification
"Pattern–score correlations were r = .25 with actual stress vulnerability, r = .13 with negative affect, and near zero with positive affect (r = .01) and extraversion (r = .00), confirming convergent and discriminant validity."
- What is meant by "actual stress vulnerability"? How do these results demonstrate convergent and discriminant validity?
"Neurosynth decoding against 50 topic maps from 11,406 fMRI studies indicated greatest similarity with the broad topic 'stimulation.'"
-How do the authors interpret this finding?
"Hence, this predictive model is a strong candidate for the neural basis of vulnerability to stress… the detected effect size corresponds to the median effect size of individual-difference research more generally."
- These sentences are difficult to interpret and need to be rewritten for clarity.
"The vulnerability pattern was further tests concerning its psychological and neurobiological interpretability."
- I think this sentence is grammatically incorrect
"Trait-like responsivity to negative stimuli was measured as the person-wise average difference in self-reported momentary affect, based on a single-item 5-point Likert scale."
- This description is confusing. Is this measure equivalent to "negative affective states based on dynamic self-reports collected during the scenes task"? If so, please clarify whether this represents a trait or a state measure.

Miquel A. Fullana

(Remarks on code availability)

Reviewer #2

(Remarks to the Author)
This study aimed to assess whether individual negative emotion dispositions, specifically neuroticism and its facets, could be predicted by amygdala and salience network activity during emotional face and scene tasks using a large community sample. A structured investigation using multiple neural measures and multiple combinations of modeling choices was conducted. Neuroticism could not be predicted from neural measures. Stress vulnerability was predicted by whole brain neural activation patterns but did not involve salience-related brain areas. An exploratory multiverse analysis indicated that emotional dispositions were more decodable when affective trait was task derived. This work addresses a gap in knowledge regarding the ability to use neural measures (especially neural signatures) to reliably predict negative emotional traits. The manuscript is clearly written and includes robust tests of reliability and validity. Data sharing, including an interactive online app, is commendable. The following comments and questions aim to further unpack the rationale, findings, and impact of this work:

-The decision to use face and scene viewing tasks was pragmatic but also seems at odds with a point made in the introduction (and elsewhere): "it is likely that neural correlates of negative affectivity can mainly be observed in appropriate task contexts, psychologically aligned with the specific construct of interest." Further elaboration on the decision to use very simple, classic tasks to assess neural mechanisms of complex emotional traits would be helpful. It seems that to make definitive claims regarding predictive capabilities for emotional traits, neural activity in tasks more closely linked to those traits should be used.

-The multiverse analysis comparing different affective traits is important and the findings that predictive accuracy conforms to a face validity gradient are intuitive. Extrapolating from this, I wonder if models trained specifically on high negative affect trials/contrasts defined using the subject's own ratings would better predict facets of neuroticism? Showing the results from models that predict task-based affective ratings could be informative since they showed the highest correlations.

-Further justification and discussion regarding scaling versus centering the data would be useful. Why does this choice seem to make a difference when correlating stress vulnerability and activity in certain regions (like the amygdala) but not in others? The Discussion indicates that centering data can address global signal biases, but why should a global bias only influence results in certain regions rather than across the whole brain? The design factors analysis (figure 6) suggests that scaling choices have the least importance compared to other design factors, but that doesn't seem to be the case in the reported results.

-The discussion primarily emphasizes how these results contradict region- and network-based findings in the field and makes methodological recommendations for future research. More description of what was found in the stress vulnerability signature and contextualizing this result in the broader literature of affective neural signatures would be useful for future work.

-Minor: it would be helpful to describe what the error bars represent in figures 2-4 as well as include a visual indicator for which correlations are significant in figures 3 and 4.

(Remarks on code availability)
My review was only cursory. The data and code seem organized and documented.

Reviewer #3

(Remarks to the Author)
I co-reviewed this manuscript with one of the reviewers who provided the listed reports. This is part of the Nature Communications initiative to facilitate training in peer review and to provide appropriate recognition for Early Career Researchers who co-review manuscripts.

(Remarks on code availability)

Reviewer #4

(Remarks to the Author)
The manuscript „The functional neurobiology of dispositions towards negative emotions" by Sicorello et al. investigates the neurobiological foundations of negative affectivity and particularly whether activity of the amygdala and salience networks, neural markers long theorized to underlie individual differences in neuroticism and related emotional dispositions, actually predict traits related to the experience of negative emotion. Using preregistered analyses of two standard emotional fMRI paradigms (i.e., face and scene processing), the authors report interesting null findings in which broad neuroticism showed no reliable association with amygdala responses, salience network activity or neural signatures. However, a distributed, whole-brain machine learning signature modestly predicted the specific facet of stress vulnerability which was replicated in independent data. Interestingly, this predictive pattern excluded regions traditionally emphasized in the literature. Further, through an extensive multiverse analysis encompassing fourteen trait constructs and over thousand models, the authors demonstrate that the decodability of emotional dispositions from neural activity depends critically on the alignment between trait and task context. As such, the authors suggest that prior research on trait negative affectivity, particularly based on resting-state fMRI, may have overestimated the role of specific neural substrates while underappreciating the distributed, context-dependent nature of brain-trait relationships.

The manuscript presents several notable strengths that substantially contribute to its scientific rigor and impact. First, the authors employ multiple levels of brain markers such as region-of-interest analyses, network-based approaches and machine learning signatures which represent the most prevalent methodological frameworks in contemporary affective neuroscience, thereby making a systematic effort to compare their relative decodability. Second, the study leverages a sizable, carefully selected community that enhances generalizability beyond typical convenience samples. Third, the investigation encompasses a comprehensive range of trait constructs related to negative affectivity, spanning from high-level broad dispositions to more specific low-level facets with population-representative variance, allowing for nuanced examination of brain-trait relationships. Fourth, the study is preregistered and the authors maintain transparency regarding deviations from the preregistration plan, enhancing reproducibility and trustworthiness of the findings. Additional methodological strengths include the use of two most commonly used emotional fMRI paradigms (face and scene processing), follow-up analyses using a Bayesian framework to provide positive evidence for null findings rather than relying solely on frequentist approaches, and actual testing of predictability in a hold-out sample rather than relying on cross-validation alone. The manuscript is well-written with a clear logical flow of ideas, and the analysis workflow is readily understandable. The inclusion of additional analyses generally follows a logical stream, though occasionally some supplementary tests appear without prior motivation in the introduction which may surprise readers who have not yet read the methods section. Finally, Figure 1 provides an excellent visual summary of the study design and aims, and the authors' implementation of a multiverse analyses and the provision of an online interactive application allowing exploration of factors influencing predictability demonstrates their commitment to open science and data accessibility.

In the following, I want to provide some comments and remarks that I hope will be of help during a revision process to increase the readability and clarity of this manuscript for the reader.

- The authors argue convincingly that brain-trait associations are context-dependent, yet there is limited discussion of how this principle applies to their own methodological choices. Specifically, while the manuscript justifies the use of face and scene processing tasks as the two most common emotional fMRI paradigms, it remains somewhat underexplored in the discussion whether those or other negative affectivity-related traits might be better engaged with different task contexts. For instance, acute stress reactivity or other facets might show stronger neural predictability under different experimental manipulations. This consideration is briefly mentioned in the introduction regarding states versus traits but could benefit from more explicit discussion, particularly given the central thesis that decodability depends on trait-task alignment.

- Similarly, the authors should consider qualifying their conclusions regarding the absence of associations with specific regions and networks by more explicitly noting that these null findings are observed within the specific context of the face and scene processing tasks employed. While these tasks are indeed the most commonly used paradigms, they are not necessarily optimal for activating all relevant regions and networks, and alternative tasks might yield different patterns. Thought their work clearly adds to a growing body of work demonstrating the limitation of direct trait-neural activiation associations, this qualification could be interesting given that distributed signatures may be inherently superior to region- or network-based approaches regardless of task choice, and distinguishing between these possibilities would strengthen the interpretability of the findings.

- Table 1 would benefit from clarification regarding whether the values presented as "+/-" represent standard deviations, standard errors, or ranges, as this is not specified in the table legend.

- Figure 2 includes the statement "Hollow circles for the best region approach indicate the point estimates from the split-half selection sample," but the meaning of this methodological detail is not adequately explained in the results section where the figure is introduced. More generally, I would recommend rereading the results section from the perspective of readers who have not yet read the methods section. In some instances, additional methodological context within the results would substantially improve comprehension and flow.

- The authors could provide the detected effect size of the predictive model for vulnerability to stress, as this could help evaluating the practical significance of the finding and for contextualizing it within the broader literature on brain-trait associations.

- The manuscript would benefit from consistent terminology throughout. Specifically, "scenes" and "IAPS" appear to be used interchangeably; selecting and maintaining a single term would reduce potential confusion. Similarly, the terms "construct" and "outcome choice" are both used in contexts where greater consistency would aid clarity (e.g., in the second paragraph of the exploratory multiverse analysis and in Figure 6).

- For the Bayesian analyses, the authors should explicitly state that tests are conducted for the null hypothesis. Readers accustomed to BF_{10} notation may initially misinterpret the given Bayes Factors, and only the notation in Table S2 clarifies that BF_{01} is being reported. Greater specificity in the main text would prevent confusion.

- The discussion section would benefit from deeper considerations with regard to theoretical implications of the findings rather than primarily restating results. Several critical questions merit substantive consideration: Are decades of prior research on the amygdala and salience network in negative affectivity now rendered obsolete, or do these findings suggest a more nuanced reinterpretation? How do the null findings for these regions square with basic neuroscience and animal model work, which often show clear involvement of the e.g., amygdala in threat processing and negative emotion? The current discussion compares findings primarily to prior fMRI work, but deeper integration with the broader neuroscience literature could in my view strengthen the manuscript. Additionally, specific findings in my opinion deserve more detailed discussion if space allows. For instance, the seemingly contrarian association between stress vulnerability and dorsal anterior cingulate cortex, dorsal attention, frontoparietal, and default mode network activity, or why dropping the somato-motor network causes the largest performance drop in the lesion analysis (this is mentioned but could be elaborated further, I think). Rather than recurrently emphasizing the limitations of region- and network-focused approaches, the discussion might more constructively position this work as part of an emerging body of evidence that is reshaping our understanding of brain-trait relationships.

- There are numerous minor typos throughout the manuscript and supplement materials which warrant correction during revision (e.g., "tests" vs. "tested" on page 8, line 165; missing closing parentheses). A careful proofreading pass would improve polish and readability.

- The addition of the NeuroSynth maps decoding approach in the psychometric assessment paragraph is interesting but appears somewhat unexpected. The motivation for including this particular analysis could be more clearly stated, and it would be helpful if the authors discussed how this approach complements or contrasts with the other analytical strategies employed.

- Supplementary Figure S1 may benefit from adjustments to line transparency to better illustrate where most trajectories cluster, as the current presentation makes it difficult to see central tendencies in the data.

- In Figure 5b, the neuroticism measures (self-report and other-report) appear to have a smaller number of models compared to other constructs. Maybe I missed it but I'm unsure how this inconsistency is explained.

(Remarks on code availability)

Version 1:

Reviewer comments:

Reviewer #1

(Remarks to the Author)
This is a much improved manuscript and the authors are to be congratulated on their revision.
I have three further comments that the authors may want to consider.

1.The authors have kindly responded to my initial comment that I could not check the preregistration and have now shared it. Thanks for this! However, after inspecting the preregistration document, I realized that there are some significant differences between, for example, the main hypothesis ("A multivariate pattern whole-brain pattern can explain variance in neuroticism") and the hypothesis that the authors state in the manuscript was preregistered ("we preregistered to first predict neuroticism and, if an accuracy threshold is not surpassed, test the predictability of the six lower order facets of neuroticism before evaluating the best model in the hold-out sample"). There is nothing wrong with deviating from the preregistered hypotheses

or analyses, but this needs to be clearly acknowledged and reported.

2. I still think that several parts of the manuscript could be improved in terms of clarity and readability. To cite only two examples from the abstract: “Evidence including Bayes factors indicated that neuroticism is not associated with any region-, network-, or machine learning-based measure, including amygdala responses. In contrast, a novel brain-wide response pattern robustly predicted the facet stress vulnerability, replicated in an independent dataset”. A “brain-wide response pattern” is often derived using machine learning, and therefore the wording sounds contradictory. “…our findings demonstrate the highly selective decodability of 43 emotional dispositions from brain responses”. I doubt that most readers not familiar with this literature will understand the meaning of “decodability”.

3. A number of other expressions and sentences in the manuscript would probably benefit from some revision: “evidence from daily life”, “withdrawal-related facets of neuroticism”, “trait realizations”, “related to approach-oriented volatility-facets”, etc.

MINOR/OTHER
“Neurosynth decoding against 50 topic maps from 11,406 fMRI studies indicated that the stress vulnerability pattern has the largest similarity with the broad topic “stimulation” (r = .26). Hence, the neural pattern might reflect a domain-general neural responsiveness to stimulation in people with 245 higher vulnerability to stress, beyond specifically affective processing”

What do the authors mean by “a domain-general neural responsiveness to stimulation”?

Use “stress vulnerability” or “vulnerability to stress”; do not use “vulnerability” alone (e.g., in Figure 3).

(Remarks on code availability)

Reviewer #2

(Remarks to the Author)
The authors have thoroughly addressed my comments and questions.

(Remarks on code availability)

Reviewer #3

(Remarks to the Author)
I co-reviewed this manuscript with one of the reviewers who provided the listed reports. This is part of the Nature Communications initiative to facilitate training in peer review and to provide appropriate recognition for Early Career Researchers who co-review manuscripts.

(Remarks on code availability)

Reviewer #4

(Remarks to the Author)
The authors have addressed all of my comments in a thorough and comprehensive manner. Their revisions demonstrate careful consideration of each of my points raised and the manuscript has been meaningfully improved in clarity. I thank the authors for their diligent work and have no further concerns.

(Remarks on code availability)

Open Access This Peer Review File is licensed under a Creative Commons Attribution 4.0 International License, which permits use, sharing, adaptation, distribution and reproduction in any medium or format, as long as you give appropriate credit to the original author(s) and the source, provide a link to the Creative Commons license, and indicate if changes were made.
In cases where reviewers are anonymous, credit should be given to 'Anonymous Referee' and the source.
The images or other third party material in this Peer Review File are included in the article’s Creative Commons license, unless indicated otherwise in a credit line to the material. If material is not included in the article’s Creative Commons license and your intended use is not permitted by statutory regulation or exceeds the permitted use, you will need to obtain permission directly from the copyright holder.
To view a copy of this license, visit https://creativecommons.org/licenses/by/4.0/

General Response:

We thank the reviewers for their time, constructive feedback and suggestions, which helped us improve the manuscript considerably in terms of structure, clarity, and content. We made comprehensive changes to the Introduction and Results sections to address these comments, also using feedback on clarity from researchers at our department unfamiliar with the project. We also made comprehensive changes to the structure of the Discussion section, now underscoring and emphasizing specific points raised by the reviewers. For more details, see our responses below. We attached versions of the manuscript with and without tracked changes, as the latter might increase the readability of some sections (e.g., the abstract).

Reviewer #1 (Remarks to the Author):

Sicorello et al. present a manuscript on the prediction (in machine-learning terms) of affective traits from neural (fMRI) data. This topic is important because it addresses the neural correlates of negative affect traits from both basic and applied perspectives. Moreover, the authors employ sophisticated and up-to-date analytical methods. Overall, the manuscript has potential, and the results (even if largely null or modest) are of interest. However, the manuscript requires substantial editing and revision before it can be considered for publication.

These are some points that the author may take in consideration. I hope this is helpful.

COMMENT R1.1
1. Clarity and conceptual precision
I found the manuscript overall difficult to follow. The abstract and introduction are confusing, and the use of several terms and concepts is imprecise. For example, the authors appear to use “depressivity” and “depression” interchangeably, and the term “prediction” is inconsistently applied. In addition, after reading the introduction, it remains unclear how the authors distinguish between trait and state constructs.

Response:

a) We revised the entire manuscript for clarity and consistency in usage of terms. Beginning with the abstract, as noted by the Reviewer, we provide a revised version (see below), which we hope is clearer. We tried to omit minor points. We also made clearer that the study focuses on task-based responses (“using neural responses in the two most common emotional fMRI tasks”).

There is one deliberate difference between the abstract and the main text in terms of terminology. In the manuscript, we use the term “negative affectivity”, while we use “dispositions towards negative emotions” in the abstract. The reasoning behind this was that for a broad multi-disciplinary audience, the latter phrase might be easier to understand when screening the abstract. At the same time, it would be overly long to use throughout the manuscript, and it is rarely used in the more specialized literature. Accordingly, we explicitly

explained our terminology (i.e., “negative affectivity”) at the beginning of the introduction in line with the Reviewer’s suggestion.

Abstract:

“People differ in their tendency to experience negative emotions. This variability is largely captured by broad psychological constructs like neuroticism, whose facets include anxiety, depression, and stress vulnerability, among others. For decades, the amygdala and salience networks have been assumed to underlie such dispositions, despite inconsistent evidence. We preregistered a comprehensive test of these hypotheses—accompanied by theory-agnostic machine learning prediction—using neural responses in the two most common emotional fMRI tasks (scenes and faces; N=338/424). Evidence including Bayes factors indicated that neuroticism is not associated with *any* region-, network-, or machine learning-based measure, including amygdala responses. In contrast, a novel brain-wide response pattern robustly predicted the facet *stress vulnerability*, replicated in an independent dataset (r=.21/.19). Predictive performance most strongly depended on somatomotor and visual networks, rather than salience-network regions, pointing to a basis for stress vulnerability in cortical perception–action systems. Together with a multiverse analysis spanning 14 trait constructs and 1,176 models, our findings demonstrate the highly selective decodability of emotional dispositions from brain responses to common affective tasks. Therein, they highlight the importance of construct and task selection, while challenging the role of the most commonly used neural markers, including responses of the amygdala and salience network.”

b) We exchanged “depressivity” for “depression” throughout the manuscript for consistency, as this is the term used by the NEO model for the corresponding facet.

c) We made the distinction between traits and states clearer by providing a separate paragraph on this distinction and its implication:

“Most of these neural targets were motivated by process-oriented studies of negative affective *states* (i.e., dynamic momentary experiences), which estimate “within-person” effects by comparing the same individuals across conditions (e.g., negative vs neutral stimuli). This literature is often taken to imply that neural systems supporting momentary negative affective states are also core substrates of negative affective *traits*, that is, “between-person” differences in relatively stable dispositions.”

d) We reviewed and verified our use of the terminology for “prediction”/”predictive” throughout the manuscript. We now only use it to describe machine learning models, where we believe the term is most appropriate, as “prediction” is routinely used when predicting an outcome from a different type of data in statistics and machine learning (i.e., without a prospective temporal aspect). For relations without regard to machine learning models, we tried to consistently use the term “association”. Notably, there are some minor exceptions, which were hard to resolve. For example, when simultaneously referring to the results of the 44 theory-based tests as “associations”, they consist of 36 bivariate correlations, but also 6 machine learning models and one multiple regression, where the term “prediction” also might be appropriate. As these multivariate models were of secondary importance, we hope this terminology is acceptable.

e) We made minor changes throughout the manuscript to increase clarity based on the Reviewer’s point. These changes were based on carefully re-reading the paper and incorporating feedback by two researchers without knowledge of the project. Some additional specific passages were pointed out by the other Reviewers as needing improvement in clarity, and we revised those passages as well.

COMMENT R1.2
2. Study goals
The exact goals of the study are not clearly stated, which may partly stem from the writing issues noted above. The manuscript should explicitly define its aims and hypotheses.

Thank you for this comment. We now explicitly state the primary goal in the Introduction as:

"The goal of this preregistered study was to comprehensively test how well negative affectivity is reflected by the three outlined neuro-organizational levels—single regions, canonical resting-state networks, and validated neural signatures (Figure 1a)—and compare them to a novel machine learning model drawing on brain-wide distributed signals"

A more precise explanation of how this aim is operationalized is provided in the subsequent paragraphs (e.g., that only task responses are used together with the respective rationale).

The preregistered hypotheses are now stated at the end of the introduction:

"In the preregistration, we hypothesized that a whole-brain predictive pattern for neuroticism would outperform region-, network-, and signature-based models. Given the proposed importance of aligning fMRI tasks with psychological constructs, we expected this pattern to (1) show greater accuracy for the scenes task than the faces task, as the latter might be targeting emotion recognition more closely than emotional experiences; (2) correlate more strongly with negative affect than with positive affect or extraversion; and (3) primarily reflect withdrawal-related facets of neuroticism, including depression, anxiety, and stress vulnerability."

In the preregistration, we described all the theory-based tests performed in the paper, without formulating an explicit hypothesis. This decision was based on the overarching goal of the study to include the best available common and novel neural markers of negative dispositions and compare them. Hence, each included neural marker is selected and tested to be a potential correlate of negative affectivity based on this formulated goal, with the implicit hypothesis being the existence of an effect. For example, we wrote in the preregistration how the association between amygdala responses and neuroticism will be tested, but not explicitly that the hypothesis is there to be an association per se, which we believed to be clear from the stated goal to test for this association.

Notably, the preregistration was structured as first explaining the machine learning analyses and then the theory-guided tests. We reversed this order in the manuscript. This was intended to make it easier to follow the manuscript, as the theory-guided tests are shorter and required fewer preregistered follow-up analyses.

COMMENT R1.3
3. Writing and style
The text needs thorough editing for grammar, syntax, and word choice (there are several typos, missing words, and grammatical errors). Some expressions are incorrect or confusing. For instance, in the sentence "In contrast to single regions and resting-state networks, affective neural signatures…", the intended comparison is unclear, because the authors are not referring to regions per se but to measures (of brain activation). Similarly, the phrase "for neural salience regions based on structure, functional connectivity, and responsivity to negative stimuli" conflates a technique (functional connectivity) with a process (responsivity).

Response:

We thoroughly reviewed the manuscript based on this comment, also using ChatGPT 5 pro to double check specifically for typesetting and spelling errors. Following this procedure, we corrected all detected editing errors throughout the manuscript. Concerning the two specific issues pointed out by the reviewer:

The first example section pointed out by the reviewer ("In contrast [...]") was replaced with the following statement. We made this change, as we received feedback that a reference to regions and networks is not necessary to introduce neural signatures:

"Besides leveraging experimental tasks, a neurobiological understanding of negative affectivity might be improved by using state-of-the-art neural markers of momentary affective states. Affective neural signatures are [...]"

For the second explicitly noted section, we tried to be more precise: "[...] based on differences in structural measures, resting-state functional connectivity, and task-evoked responses to negative stimuli".

COMMENT R1.4
4. Presentation of results and figures
The presentation of the results—including the figures—needs major improvement.
Figure 1: It is unclear that fMRI data are used; "regions" is ambiguous (resting-state or task-based?), and the sample is not described. The meaning of "added choices" and "Six sub-facets" is also unclear.
Figure 2: The acronyms "PINES" and "VIFS" are never defined.
Figure 3: The reader first encounters "virtual lesion" results without any prior explanation of the term. Some results ("Other self-report" and "combined.") also are new for the reader.

Response:

Figure 1:

We tried to be more precise by writing in the figure legend (changes in italics):

"Theory-driven analyses *targeting task-based fMRI responses of anatomical brain* regions, *canonical resting-state* networks, and *affective neural* signatures". The sample is now described in the same paragraph of the introduction in which Figures 1a and 1b are first introduced. Further information on the sample is provided in Table 1 instead of the figure, referred to in the same paragraph. This paragraph now also states the sample size:

"The goal of this preregistered study was to comprehensively test how well negative affectivity is reflected by the three outlined neuro-organizational levels—single regions, canonical resting-state networks, and validated neural signatures (Figure 1a)—and compare them to a novel machine learning model drawing on brain-wide distributed signals. All neural measures were based on responses to negative stimuli in the most widely used affective fMRI task: viewing pictures of negative faces and scenes (Figures 1b). This mirrors the theoretical assumption that negative affectivity results from generally heightened neural responsivity to negative stimuli and events[3,12,14] and enhances the generalizability to the majority of the remaining task-based fMRI

literature on negative affectivity. These analyses were performed on a large community sample (*N*=338/424; Table 1) with representative variance on neuroticism based on U.S. population norms[47] (for descriptive statistics and correlations between all negative affectivity measures, see Table 2)."

The terms of the machine learning strategy (Figure 1c) are now explained in more detail:

"Each stage adds modeling choices to the previous stages (e.g., additional outcomes, algorithms, or using the full data). The additional outcomes of stage 2 are strongly linked to neuroticism. "Six Sub-Facets" includes the facets of neuroticism: depression, anxiety, stress vulnerability, anger, impulsiveness, and self-consciousness. "Other-reports" refer to neuroticism scores provided by informants, while "Self-Other-Reports" combine these with traditional self-reports (see methods section for details)."

Figure 2:

We added the definitions of the acronyms to the figure legend, which were unfortunately only provided in figure 1.

Figure 3:

Virtual lesions are now noted in the main text when referring to the respective figure 3b, including a literature reference:

"Removing single regions or networks with virtual lesion analyses[55]".

"Other reports" have now been explained more explicitly in figure 1 (see changes above). The wording in figure legend 3b has been slightly changed: "self-reports vs reports of close informants vs the combination of both (see methods section)"

COMMENT R1.5

5. Preregistration
A key strength of the study would be preregistration of its aims and hypotheses. However, I could not see this information in the link provided by the authors in page 5 (In fact, I could not see any preregistration document)

Response:

We apologize for the inconvenience! We could verify the access to the preregistration file in the linked repository, which can be downloaded from the page. Unfortunately, GitHub does not support viewing the preregistration without downloading it. GitHub was chosen over OSF to organize all materials in one place, which we see now has clear downsides for usability. We now include a link to the specific file in the manuscript (rather than the repository), mentioning that it can be downloaded there. The link is: https://github.com/MaurizioSicorello/NeuroSquare_repo/blob/main/Preregistration.docx

We checked the link with different browsers and machines and hope it is now more accessible. It can be downloaded by clicking on "View raw" in the center of the screen or by using the download icon on the upper right (under the time stamp "6 years ago", tracking the last change of the file).

COMMENT R1.6
6. Sample description
The authors state in the abstract that the results are based on "a large community sample with population-representative variance in the targeted traits." Yet, neither the Participants section nor Table 1 clearly supports this claim. In particular, descriptive statistics for the questionnaire scores are missing from the main text.

Response:

We are now more specific that this statement is based on neuroticism scores compared to U.S. population norms, providing the reference for the norm sample. We also moved the table with descriptive statistics and correlations between negative affectivity measures to the main text (i.e., Table S1 is now Table 2):

"These analyses were performed on a large community sample (N=338/424; Table 1) with representative variance on neuroticism based on U.S. population norms[47] (for descriptive statistics and correlations between all negative affectivity measures, see Table 2)."

COMMENT R1.7
7. Specific issues requiring clarification

"Pattern–score correlations were r = .25 with actual stress vulnerability, r = .13 with negative affect, and near zero with positive affect (r = .01) and extraversion (r = .00), confirming convergent and discriminant validity."
- What is meant by "actual stress vulnerability"? How do these results demonstrate convergent and discriminant validity?

Response:

We removed the "actual", as it was simply a correlation with stress vulnerability.
We replaced "confirming convergent and discriminant validity" with: "providing evidence for convergent and discriminant validity (i.e., positive correlations to related constructs and negligible correlation to conceptually distinct constructs)".

COMMENT R1.8
"Neurosynth decoding against 50 topic maps from 11,406 fMRI studies indicated greatest similarity with the broad topic 'stimulation.'"
-How do the authors interpret this finding?

Response:

We added the following

"Hence, the neural pattern might reflect a domain-general neural responsiveness to stimulation in people with higher vulnerability to stress, beyond specifically affective processing. Similarly, spatial correlations between the vulnerability pattern and the five theory-driven affective signatures were all < |r| = .02, indicating the stress vulnerability signature captures something distinct from momentary emotional states."

COMMENT R1.9

"Hence, this predictive model is a strong candidate for the neural basis of vulnerability to stress... the detected effect size corresponds to the median effect size of individual-difference research more generally."
- These sentences are difficult to interpret and need to be rewritten for clarity.

Response:

We changed the sentences to (respectively):
"Thus, the whole-brain prediction model reliably generalized to new participants, indicating a robust brain–trait mapping for vulnerability to stress and a first step in determining a potential neural marker"

"Notably, the detected correlation corresponds to the median correlation of meta-analytic individual difference associations across research fields[56]"

COMMENT R1.10
"The vulnerability pattern was further tests concerning its psychological and neurobiological interpretability."
- I think this sentence is grammatically incorrect

Response:
The error was corrected.

COMMENT R1.11
"Trait-like responsivity to negative stimuli was measured as the person-wise average difference in self-reported momentary affect, based on a single-item 5-point Likert scale."
- This description is confusing. Is this measure equivalent to "negative affective states based on dynamic self-reports collected during the scenes task"? If so, please clarify whether this represents a trait or a state measure.

Response:
In this sentence, we equated "traits" with "individual differences", which was imprecise. Also, we did not state the difference between what was measured. We tried to clarify both in the sentence below (additions in italics). Overall, we believe these task-based measures to have both state- and trait-like components, with unclear mixture.

~~"Trait-like~~ *Individual differences in task-based* responsivity to negative stimuli were measured as the person-wise average difference in self-reported momentary affect *between negative and neutral experimental trials*, based on a single-item 5-point Likert scale (see task description for further information)"

Miquel A. Fullana

Reviewer #2 (Remarks to the Author):

This study aimed to assess whether individual negative emotion dispositions, specifically neuroticism and its facets, could be predicted by amygdala and salience network activity during emotional face and scene tasks using a large community sample. A structured investigation using multiple neural measures and multiple combinations of modeling choices was conducted. Neuroticism could not be predicted from neural measures. Stress vulnerability was predicted by whole brain neural activation patterns but did not involve salience-related brain areas. An exploratory multiverse analysis indicated that emotional dispositions were more decodable when affective trait was task derived. This work addresses a gap in knowledge regarding the ability to use neural measures (especially neural signatures) to reliably predict negative emotional traits. The manuscript is clearly written and includes robust tests of reliability and validity. Data sharing, including an interactive online app, is commendable. The following comments and questions aim to further unpack the rationale, findings, and impact of this work:

COMMENT R2.1

-The decision to use face and scene viewing tasks was pragmatic but also seems at odds with a point made in the introduction (and elsewhere): "it is likely that neural correlates of negative affectivity can mainly be observed in appropriate task contexts, psychologically aligned with the specific construct of interest." Further elaboration on the decision to use very simple, classic tasks to assess neural mechanisms of complex emotional traits would be helpful. It seems that to make definitive claims regarding predictive capabilities for emotional traits, neural activity in tasks more closely linked to those traits should be used.

Response:

There were two main reasons for choosing these tasks. Firstly, most theories concerning negative affectivity assume that high-scorers have a generally heightened neural reactivity to negative stimuli across contexts. The most influential examples are Eysenck's neuroticism theory and Linehan's theory on emotional instability in Borderline Personality Disorder. Therefore, if correct, these two classic tasks should probe this idea of context-generality, which pervades large parts of theory and clinical research. Secondly, we wanted our results to be informative on large parts of the published literature (therefore, being able to consolidate, compare, contextualize, and expand what can be learned from this vast literature that often relies on these paradigms). Hence, these were the task paradigms most commonly used in personality and clinical fMRI research to make claims concerning individual differences in affective functioning. Based on the literature, these tasks could be considered strong candidates. Although we had a degree of skepticism concerning their utility, there was, to our knowledge, no other candidate task which was clearly superior (especially to warrant assessment in such a relatively large sample, which is costly but needed for meaningful fMRI analyses on individual differences). We do agree with the reviewer that more explanation concerning these issues is important for the paper.

In the introduction, we added (in italics):

"All neural measures were based on responses to negative stimuli in the most widely used affective fMRI task: viewing pictures of negative faces and scenes (Figures 1b). *This mirrors the theoretical assumption that negative affectivity results from generally heightened neural responsivity to negative stimuli and events across contexts*[3,12,14] *and enhances the generalizability to the majority of the remaining task-based fMRI literature on negative affectivity."*

In the discussion, we explained the task choice in more detail within a new paragraph:

"Our preregistered machine learning analyses highlight that not all facets of negative affectivity are equally decodable from negative affective scenes and faces. Anticipating that these tasks are less related to approach-oriented volatility-facets (impulsiveness, angry hostility), we preregistered the qualitative expectation of higher decodability for withdrawal-related facets (stress-vulnerability, depression, anxiety, self-consciousness). Still, the multiverse analysis showed only a predictive advantage of stress vulnerability, not the remaining withdrawal-facets, which was also only apparent for the scenes task. This is highly plausible as the psychological definition of stress vulnerability mirrors the scenes task: How individuals respond to negative events. In contrast, depression is more focused on prolonged affective states and anxiety on the anticipation of threat. Therefore, qualitatively different tasks might better target those facets, like ruminative state switching[69] and threat-of-shock[70], respectively. We nevertheless focused on negative scenes and face viewing because they are the most widely used affective fMRI tasks and are often treated as broadly applicable probes of 'general' negative responsivity, consistent with biological theories of negative affectivity[3,12,14,35]. Using these canonical tasks therefore provides a stringent and maximally field-relevant test of the context-generality assumption, which still underlies current large-scale studies[71]. At the same time, our findings do not exclude that other tasks could yield more accurate predictions of negative affectivity facets or even broad neuroticism. Identifying robust facet-selective task contexts remains an open methodological need for the field[1,72,73]. Importantly, one possibility is that broad negative affectivity is "the sum" of facet-specific mechanisms that are each most visible in different, facet-selective task contexts. If so, higher-order traits like neuroticism may only yield coherent neural correlates when aggregating across a battery of such tasks, also clarifying which psychological constructs have a consistent neurobiological counterpart."

COMMENT R2.2

-The multiverse analysis comparing different affective traits is important and the findings that predictive accuracy conforms to a face validity gradient are intuitive. Extrapolating from this, I wonder if models trained specifically on high negative affect trials/contrasts defined using the subject's own ratings would better predict facets of neuroticism? Showing the results from models that predict task-based affective ratings could be informative since they showed the highest correlations.

Response:

This is a very interesting point! In discussions, we often experienced two contrasting hypotheses. One hypothesis is that maximally strong within-person contrasts (e.g., high negative vs neutral) lead to the best neural measures for individual differences. Another hypothesis, from classical test theory, would assume that an average score based on different item difficulties is best suited. In our case, items correspond to different stimuli and moderately negative (rather than extremely negative) stimuli should differentiate best among participants. So far, we are not aware of a study which systematically evaluated this.

We tested the suggested analyses, although we used stress vulnerability instead of average task-based affective ratings. We agree with the reviewer that the latter are generally the more robust target for decoding analyses. Still, for the specifically suggested analyses, the average self-ratings contain information from all trials and the neural responses from moderate trials are systematically more similar to these overall self-report averages than maximally intense trials.

We added to the manuscript:
"Lastly, in post-hoc analyses we observed that the prediction of stress vulnerability was better when using only highly intense trials (rating = 5, r = .05) than moderately intense trials (rating = 3, r = .00), although both models were well-below the results for averaging all trials. This might point towards the relevance of both the number of trials and the intensity of stimuli."

We already planned a more comprehensive analysis on this question (maximum intensity versus test theoretically derived stimuli) for a follow-up study with more suitable data and hope these analyses are sufficient for the present manuscript.

COMMENT R2.3

-Further justification and discussion regarding scaling versus centering the data would be useful. Why does this choice seem to make a difference when correlating stress vulnerability and activity in certain regions (like the amygdala) but not in others? The Discussion indicates that centering data can address global signal biases, but why should a global bias only influence results in certain regions rather than across the whole brain? The design factors analysis (figure 6) suggests that scaling choices have the least importance compared to other design factors, but that doesn't seem to be the case in the reported results.

Response:

This is a very interesting question, which we also have discussed internally, while being hesitant in adding more information to the paper at first. In general, we employed these choices based on their successful use in the state-oriented MVPA literature. We observed that both centering and scaling images are common, but had no strong prior belief concerning the optimal choice between the two.

Concerning the first specific question, why centering only affects certain regions, we do not have an entirely certain answer. One hypothesis we considered was that certain regions and networks are larger and therefore overrepresented in the average signal, leading to a smaller difference between (un-)centered data. This is certainly true for the neural signatures, which contain loadings from most parts of the brain and are therefore unaffected by centering. The other unaffected regions, the dorsal attention network (including dACC), default mode network, and frontoparietal network, make up "only" 50% of the brain, so in these cases this explanation seems less likely. Another potential explanation we tested is: Responses in the dACC are more strongly correlated with global responses (r=.80) than amygdala responses (r=.64). Whether this is the case by chance or neuroanatomical influences are unclear. Generally, if a real effect exists for a given region or network, which is biased by global signal effects, the effect size should increase in the respective direction of the true effect. If no true effect exists, it is unclear what centering might do to the between-person correlation. In some cases, this might lead to no changes in correlations simply by chance, as for the dACC and strongly correlated networks. We checked that there are plausible correlation patterns between an outcome (e.g., stress

vulnerability), a predictor (e.g., dACC), and a covariate (e.g., global signal), which lead to almost no changes in the initial outcome-predictor correlation after removing the covariate, without substantive interpretation. We added the following paragraph to the manuscript:

"This robustness to centering was not explained by higher correlations of global signal responses with the three networks and the dACC. Rather, this lack of change is an algebraically plausible case given the covariance structure between stress vulnerability, global signal responses, and the four uncentered neural measures. Global signal responsiveness was overall slightly negatively correlated with vulnerability to stress, speaking against it being due to higher arousal in people with higher stress vulnerability (r = -.08, 95% CI = [-.19, .02], p = .123)."

Concerning the question why centering was not relevant for the machine learning analyses: We believe this is the case as the three most successful algorithms (PLS, PCR, SVR) are very likely to implicitly account for such effects. We now explicitly tested this option using the main algorithm (PLS) and could largely confirm this hypothesis. Although we consider this a relevant detail, we added most information concerning this issue to the supplements, due to the very large number of presented results. In the main text, we only write: "The observation that centering choice did not affect accuracy, likely indicates that these three algorithms implicitly account for global signal confounding, similar to the manual removal performed for the amygdala (see section: Combining theory- and data-driven insights). This is demonstrated in Figures S4-S5."

The two figures in the supplements then contain the in-depth information in the descriptions:

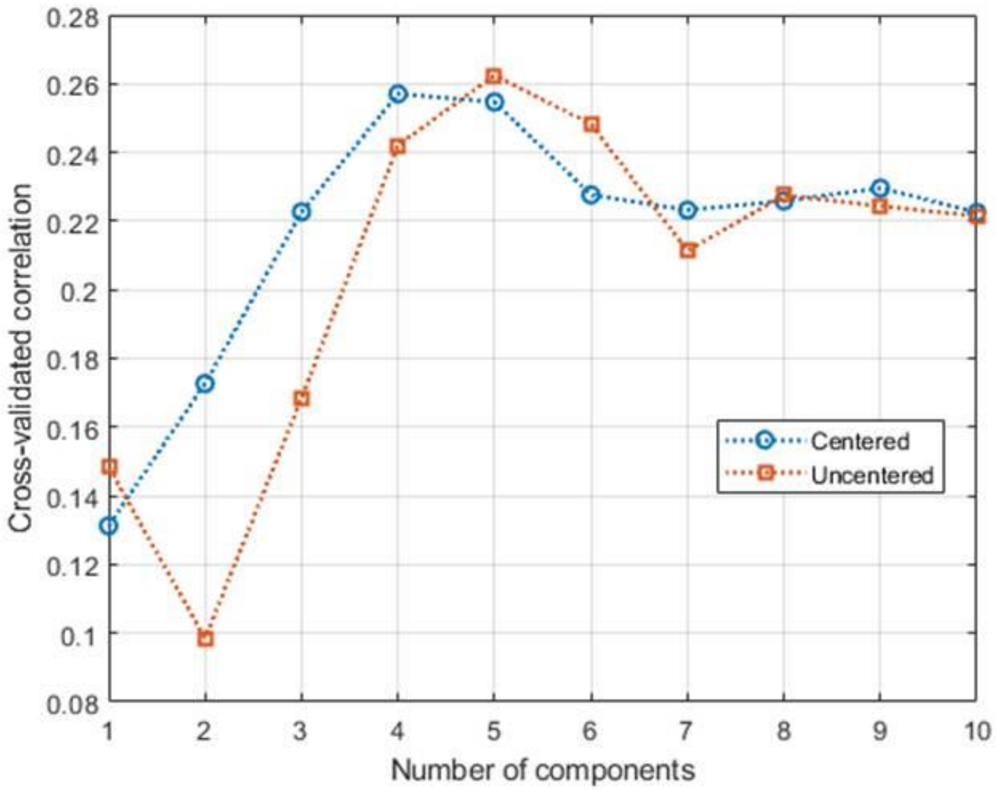

Figure S4.

Task-based affective self-reports during the scenes task were predicted from partial least squares (PLS) models with an increasing number of components. This figure shows the cross-validated correlations between predicted and actual responses as a function of the number of components in

centered and uncentered contrast images, respectively. Centering refers to subtracting the average whole-brain response of each participant from all voxels in their respective contrast image. This plot shows that PLS models on uncentered data perform equally well as on centered data after 4 components. Still, the model on centered data needs less components to reach a performance plateau. This pattern fits the hypothesis that PLS on uncentered data first has to remove confounding by whole-brain responses before "catching up" to the PLS model on centered data, where these nuisance signals are already removed. Further evidence for this explanation is provided in Figure S5.

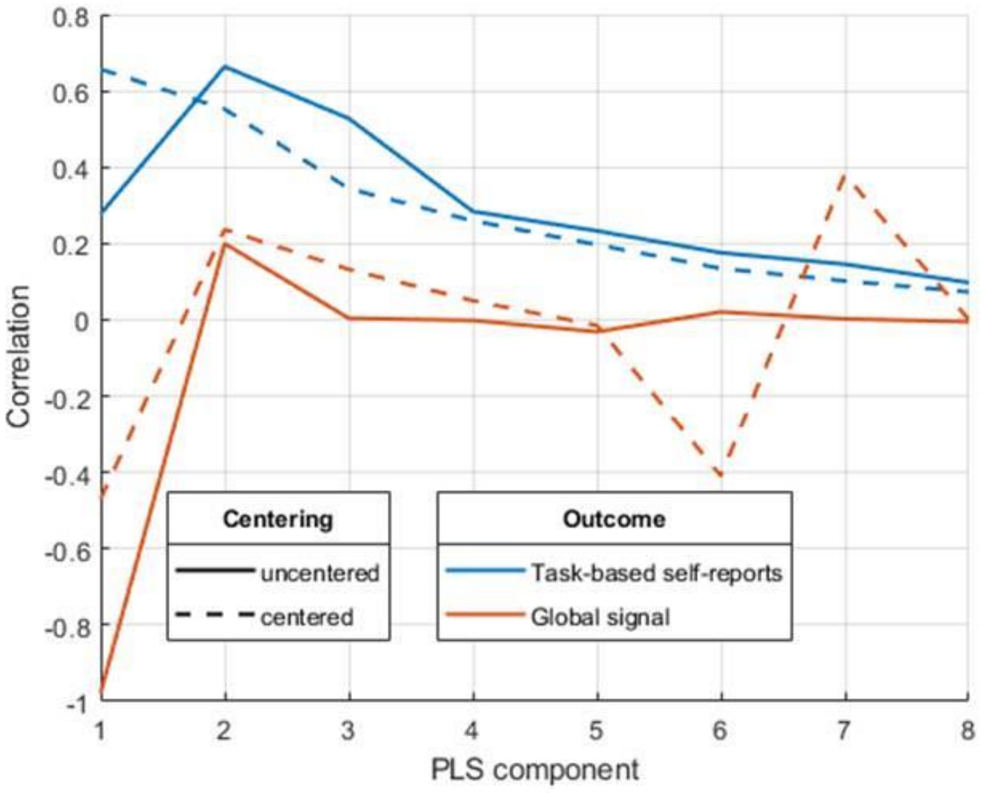

Figure S5.

Correlations (y-axis) between participants' component scores on the first eight PLS components (x-axis) and either task-based self-reports (blue) or global signal responses (red). For the PLS model on centered data, the correlation between the component scores and task-based self-reports is monotonically decreasing (dashed blue line). This is consistent with the idea that this model can extract useful information from the data already starting with the first component. For uncentered data, on the other hand, the first component has a relatively low correlation with the predicted task-based reports, rising afterwards, indicating the first component is largely capturing noise (solid blue line). This is further supported by correlations between PLS component scores and global signals responses. The first component of the uncentered model is almost perfectly negatively correlated with global signal responses. Hence, overall, these patterns of Figure S4 and S5 confirm that PLS on uncentered data is implicitly removing global signal responses and therefore performs similarly to PLS on centered data, although it might need slightly more components.

Three additional observations might be relevant for this pattern. Firstly, Figures S4 and S5 both indicated that the first PLS component of the uncentered model already had some predictive value. Follow-up analyses showed that this is the case because the task-based affective responses are correlated with the global signal responses at r = -.14. Hence, people who responded more strongly to stimuli based on self-reports actually had *smaller* whole-brain responses. This speaks against the

explanation that whole-brain responses might capture task-related hyperarousal to stimulation. Secondly, for the uncentered model, only the first two components were related to global signal. For the following components, it is perfectly removed (solid red line). In contrast, for the centered model, later components were again related to global signals, indicating that implicit PLS denoising might lead to cleaner solutions than manual denoising by image centering. Thirdly, these implicit mechanisms should generalize to PCR and SVR, but not random forest regression, as they all share an implicit similarity to L2 regularization.

COMMENT R2.4

-The discussion primarily emphasizes how these results contradict region- and network-based findings in the field and makes methodological recommendations for future research. More description of what was found in the stress vulnerability signature and contextualizing this result in the broader literature of affective neural signatures would be useful for future work.

Response:

We now expand much more on the potential theoretical implications of the vulnerability pattern in the discussion, also noting that lesioning the visual network produced similar performance reductions as for the somatomotor network:

"In contrast to broad neuroticism, vulnerability to stress was robustly predictable from task-evoked brain responses and replicated out-of-sample. Virtual lesion analyses indicated that prediction relied most strongly on visual and somatomotor systems, consistent with a distributed perception–action profile rather than localized "affective hotspot" reactivity. In line with this, the pattern showed little dependence on canonical affective regions or networks and was largely orthogonal to validated neural signatures of momentary affect, suggesting that it does not primarily indicate affect intensity. This agrees with the observation that task-based affective reports were unrelated to all negative affectivity constructs (Table 2), as previously observed for clinical negative affectivity[35]. Instead, converging evidence implicates the occipital visual cortex in encoding emotionally diagnostic stimulus information and somatomotor systems in embodied, action-oriented representations of emotional meaning[63–67]. Together, these findings are consistent with the possibility that individuals high versus low in stress vulnerability differ less in core affective feelings elicited by non-personalized laboratory stimuli than in how they construe and prepare to respond to aversive events—i.e., in the perception- and action-related relevance assigned to negative scenes. Such individual differences in affective conceptualizations might be more generalizable across experimental tasks, while an additional contribution of other regions could still emerge if personally relevant stressors are ascribed meaning[68]."

COMMENT R2.5

-Minor: it would be helpful to describe what the error bars represent in figures 2-4 as well as include a visual indicator for which correlations are significant in figures 3 and 4.

Response:

We added the descriptions of error bars and boxplot whiskers (figures 2-4) to the respective captions. In figure 4, we added significance indicators. In figure 3d, we added a dashed line indicating the significance threshold for the lesion results. The boxplots in figure 3a/b show the distribution of model accuracies across modelling choices for different constructs. In essence, they represent subsets of the multiverse analysis, for which valid straight-forward significance

tests are not available due to the complex dependencies between accuracy estimates. Therefore we refrained from inferential statements concerning these subplots: “These analyses were only performed with descriptive rather than inferential statistics, as the latter might be incorrect due to complex dependencies in the data[78]”

Reviewer #4 (Remarks to the Author):

The manuscript „The functional neurobiology of dispositions towards negative emotions" by Sicorello et al. investigates the neurobiological foundations of negative affectivity and particularly whether activity of the amygdala and salience networks, neural markers long theorized to underlie individual differences in neuroticism and related emotional dispositions, actually predict traits related to the experience of negative emotion. Using preregistered analyses of two standard emotional fMRI paradigms (i.e., face and scene processing), the authors report interesting null findings in which broad neuroticism showed no reliable association with amygdala responses, salience network activity or neural signatures. However, a distributed, whole-brain machine learning signature modestly predicted the specific facet of stress vulnerability which was replicated in independent data. Interestingly, this predictive pattern excluded regions traditionally emphasized in the literature. Further, through an extensive multiverse analysis encompassing fourteen trait constructs and over thousand models, the authors demonstrate that the decodability of emotional dispositions from neural activity depends critically on the alignment between trait and task context. As such, the authors suggest that prior research on trait negative affectivity, particularly based on resting-state fMRI, may have overestimated the role of specific neural substrates while underappreciating the distributed, context-dependent nature of brain-trait relationships.

The manuscript presents several notable strengths that substantially contribute to its scientific rigor and impact. First, the authors employ multiple levels of brain markers such as region-of-interest analyses, network-based approaches and machine learning signatures which represent the most prevalent methodological frameworks in contemporary affective neuroscience, thereby making a systematic effort to compare their relative decodability. Second, the study leverages a sizable, carefully selected community that enhances generalizability beyond typical convenience samples. Third, the investigation encompasses a comprehensive range of trait constructs related to negative affectivity, spanning from high-level broad dispositions to more specific low-level facets with population-representative variance, allowing for nuanced examination of brain-trait relationships. Fourth, the study is preregistered and the authors maintain transparency regarding deviations from the preregistration plan, enhancing reproducibility and trustworthiness of the findings. Additional methodological strengths include the use of two most commonly used emotional fMRI paradigms (face and scene processing), follow-up analyses using a Bayesian framework to provide positive evidence for null findings rather than relying solely on frequentist approaches, and actual testing of predictability in a hold-out sample rather than relying on cross-validation alone. The manuscript is well-written

with a clear logical flow of ideas, and the analysis workflow is readily understandable. The inclusion of additional analyses generally follows a logical stream, though occasionally some supplementary tests appear without prior motivation in the introduction which may surprise readers who have not yet read the methods section. Finally, Figure 1 provides an excellent visual summary of the study design and aims, and the authors' implementation of a multiverse analyses and the provision of an online interactive application allowing exploration of factors influencing predictability demonstrates their commitment to open science and data accessibility.

In the following, I want to provide some comments and remarks that I hope will be of help during a revision process to increase the readability and clarity of this manuscript for the reader.

COMMENT R4.1

- The authors argue convincingly that brain-trait associations are context-dependent, yet there is limited discussion of how this principle applies to their own methodological choices. Specifically, while the manuscript justifies the use of face and scene processing tasks as the two most common emotional fMRI paradigms, it remains somewhat underexplored in the discussion whether those or other negative affectivity-related traits might be better engaged with different task contexts. For instance, acute stress reactivity or other facets might show stronger neural predictability under different experimental manipulations. This consideration is briefly mentioned in the introduction regarding states versus traits but could benefit from more explicit discussion, particularly given the central thesis that decodability depends on trait-task alignment.

Response:

This is a very important point, similarly raised by reviewer 2. We made a change in the Introduction section to make our rationale clearer and then expanded more on this issue in the Discussion section. Notably, these changes were also made with respect to the next comment. Here are the three most important changed sections:

In the introduction, we added (in italics):

"All neural measures were based on responses to negative stimuli in the most widely used affective fMRI task: viewing pictures of negative faces and scenes (Figures 1b). *This mirrors the theoretical assumption that negative affectivity results from generally heightened neural responsivity to negative stimuli and events across contexts[3,12,14] and enhances the generalizability to the majority of the remaining task-based fMRI literature on negative affectivity."*

In the discussion, we explained the task choice in more detail within a new paragraph:

"Our preregistered machine learning analyses highlight that not all facets of negative affectivity are equally decodable from negative affective scenes and faces. Anticipating that these tasks are less related to approach-oriented volatility-facets (impulsiveness, angry hostility), we preregistered the qualitative expectation of higher decodability for withdrawal-related facets (stress-vulnerability, depression, anxiety, self-consciousness). Still, the multiverse analysis showed only a predictive advantage of stress vulnerability, not the remaining withdrawal-facets, which was also only apparent for the scenes task. This is highly plausible as the psychological definition of stress vulnerability mirrors the scenes task: How individuals respond to negative

events. In contrast, depression is more focused on prolonged affective states and anxiety on the anticipation of threat. Therefore, qualitatively different tasks might better target those facets, like ruminative state switching[69] and threat-of-shock[70], respectively. We nevertheless focused on negative scenes and face viewing because they are the most widely used affective fMRI tasks and are often treated as broadly applicable probes of 'general' negative responsivity, consistent with biological theories of negative affectivity[3,12,14,35]. Using these canonical tasks therefore provides a stringent and maximally field-relevant test of the context-generality assumption, which still underlies current large-scale studies[71]. At the same time, our findings do not exclude that other tasks could yield more accurate predictions of negative affectivity facets or even broad neuroticism. Identifying robust facet-selective task contexts remains an open methodological need for the field[1,72,73]. Importantly, one possibility is that broad negative affectivity is "the sum" of facet-specific mechanisms that are each most visible in different, facet-selective task contexts. If so, higher-order traits like neuroticism may only yield coherent neural correlates when aggregating across a battery of such tasks, also clarifying which psychological constructs have a consistent neurobiological counterpart."

"Importantly, the null findings for canonical affective systems do not invalidate their role in dynamic affective states, as they reliably respond to the tasks employed in the present study[21,23,71]. Rather, their explanatory capability for negative affectivity traits is subject to scientific boundary conditions, comprising the discussed assumption of relative context-generality across tasks and an appropriate analytical strategy."

COMMENT R4.2

- Similarly, the authors should consider qualifying their conclusions regarding the absence of associations with specific regions and networks by more explicitly noting that these null findings are observed within the specific context of the face and scene processing tasks employed. While these tasks are indeed the most commonly used paradigms, they are not necessarily optimal for activating all relevant regions and networks, and alternative tasks might yield different patterns. Thought their work clearly adds to a growing body of work demonstrating the limitation of direct trait-neural activiation associations, this qualification could be interesting given that distributed signatures may be inherently superior to region- or network-based approaches regardless of task choice, and distinguishing between these possibilities would strengthen the interpretability of the findings.

Response:

We thank the reviewer for this suggestion. Kindly see the response/changes for the last comment. In addition to this more detailed discussion on the role of task content, we also tried to mention in other sections that using these two tasks mirrors the assumption of "context-generality", i.e., the idea that heightened responsivity underlying negative affectivity can be observed for a large range of negative stimuli.

COMMENT R4.3

Table 1 would benefit from clarification regarding whether the values presented as "+/-" represent standard deviations, standard errors, or ranges, as this is not specified in the table legend.

Response:
We added the explanation that these are standard deviations.

COMMENT R4.4

- Figure 2 includes the statement "Hollow circles for the best region approach indicate the point estimates from the split-half selection sample," but the meaning of this methodological detail is not adequately explained in the results section where the figure is introduced. More generally, I would recommend rereading the results section from the perspective of readers who have not yet read the methods section. In some instances, additional methodological context within the results would substantially improve comprehension and flow.

Response:

We thank the reviewer for this observation. The original preprint was written in a "introduction -> methods -> results"-structure and restructuring might have left relevant gaps to easily follow the manuscript. As suggested, we re-read the results and tried to provide more information from the methods section to make it easier to follow. For example, the results now start with the following description:

"We first tested the association between neuroticism and neural responses across regions, networks, and validated neural signatures (Figure 1a). The region approaches comprised average responses and local machine learning patterns in three theory-derived regions (amygdala, anterior insula, and dACC). It also included a brain-wide "best region" approach, where the region most highly correlated to neuroticism is identified in one half of the data and then tested in the other half. The network approaches comprised average responses in seven major canonical networks[51] as well as linear and non-linear combinations of network responses, based on neural network theories of emotion[52]. Lastly, we included five well-validated neural signatures targeting negative affect (PINES)[21], fear (VIFS)[23], and three discrete negative emotions developed for simultaneous emotion classification (fear, anger, sadness)[53]."

COMMENT R4.5

- The authors could provide the detected effect size of the predictive model for vulnerability to stress, as this could help evaluating the practical significance of the finding and for contextualizing it within the broader literature on brain-trait associations.

Response:

We report the effect sizes in the results section together with a frame of reference:

"Here, the best model predicted the neuroticism facet vulnerability to stress at r = .21 (p = .009; one-sided) from the scenes task and the contrast [negative – neutral] using SVR and image-wise centering. In the hold-out sample, this correlation was still statistically significant (one-sided): r(100) = .19, p = .028, BF10 = 2.53, 95% CI [-.00, .37]. Thus, the whole-brain prediction model reliably generalized to new participants, indicating a robust brain–trait mapping for vulnerability to stress and a first step in determining a potential neural marker. Neuroticism, in turn, could not be decoded from responses in the two tasks. Notably, the detected correlation corresponds to the median correlation of meta-analytic individual difference associations across research fields[56]. This highlights that the often extremely high effect sizes from within-person fMRI

decoding might not be a suitable frame of reference to evaluate the relevance of between-person effects."

COMMENT R4.6

- The manuscript would benefit from consistent terminology throughout. Specifically, "scenes" and "IAPS" appear to be used interchangeably; selecting and maintaining a single term would reduce potential confusion. Similarly, the terms "construct" and "outcome choice" are both used in contexts where greater consistency would aid clarity (e.g., in the second paragraph of the exploratory multiverse analysis and in Figure 6).

Response:

We changed "IAPS task" for "scenes task" in all instances and used the term "construct" throughout. The term "outcome" is still used in some instances, but mainly to clarify a variable's position in a regression structure (e.g., "construct X served as the outcome"). We re-read the paper for consistent terminology and also had independent correctors point out to us where such inconsistencies might lead to confusion.

COMMENT R4.7

- For the Bayesian analyses, the authors should explicitly state that tests are conducted for the null hypothesis. Readers accustomed to BF_{10} notation may initially misinterpret the given Bayes Factors, and only the notation in Table S2 clarifies that BF_{01} is being reported. Greater specificity in the main text would prevent confusion.

Response:

We thank the reviewer for this important point and tried to explicitly use the BF_{01} notation in the main paper and state at the first reference what this means:

"even uncorrected Bayes factors mostly favored the null hypothesis over the existence of a true effect with odds above BF_{01} = 10 (BF_{01} = null hypothesis in the numerator; higher values are expected after multiplicity correction[54])"

COMMENT R4.8
- The discussion section would benefit from deeper considerations with regard to theoretical implications of the findings rather than primarily restating results. Several critical questions merit substantive consideration: Are decades of prior research on the amygdala and salience network in negative affectivity now rendered obsolete, or do these findings suggest a more nuanced reinterpretation? How do the null findings for these regions square with basic neuroscience and animal model work, which often show clear involvement of the e.g., amygdala in threat processing and negative emotion? The current discussion compares findings primarily to prior fMRI work, but deeper integration with the broader neuroscience literature could in my view strengthen the manuscript. Additionally, specific findings in my opinion deserve more detailed discussion if space allows. For instance, the seemingly contrarian association between stress vulnerability and dorsal anterior cingulate cortex, dorsal attention, frontoparietal, and default mode network activity, or why dropping the somato-motor network causes the largest performance drop

in the lesion analysis (this is mentioned but could be elaborated further, I think). Rather than recurrently emphasizing the limitations of region- and network-focused approaches, the discussion might more constructively position this work as part of an emerging body of evidence that is reshaping our understanding of brain-trait relationships.

Response:

Thank you. We appreciate that the first comment is aligned with the critique of the Discussion by Reviewer 2, which together made us comprehensively rework this section. We tried to focus more on contextualization and less on details that were already addressed in the results section.

Concerning integration with basic affective neuroscience and animal studies:

While we pointed out certain shortcomings of classic neural markers of affective processing in the introduction ("Moreover, even at the state level, these salience-related regions and networks show only moderate sensitivity and limited specificity for negative affective experiences[11,17–27]"), we do not believe that our study is suited to inform the basic/universal neurobiology of negative affective states in either human or animal studies (e.g., the amygdala's role in affective processing). We added the following statement to the beginning of a paragraph in the discussion to address the question raised by the reviewer:

"Importantly, the null findings for canonical affective systems do not invalidate their role in dynamic affective states, as they reliably respond to the tasks employed in the present study[21,23,71]. Rather, their explanatory capability for negative affectivity traits is subject to scientific boundary conditions, comprising the discussed assumption of relative context-generality across tasks and an appropriate analytical strategy."

The three theoretical implications we focus most on now are (1) the noted assumption of context-generality (i.e., the neurobiology of negative affectivity manifests in heightened responses to negative stimuli without topical boundary conditions) and (2) the meaning of the neural stress vulnerability pattern and the likely reasons this facet was successfully decodable. The most important changed sections can be found below:

"In contrast to broad neuroticism, vulnerability to stress was robustly predictable from task-evoked brain responses and replicated out-of-sample. Virtual lesion analyses indicated that prediction relied most strongly on visual and somatomotor systems, consistent with a distributed perception–action profile rather than localized "affective hotspot" reactivity. In line with this, the pattern showed little dependence on canonical affective regions or networks and was largely orthogonal to validated neural signatures of momentary affect, suggesting that it does not primarily indicate affect intensity. This agrees with the observation that task-based affective reports were unrelated to all negative affectivity constructs (Table 2), as previously observed for clinical negative affectivity[35]. Instead, converging evidence implicates the occipital visual cortex in encoding emotionally diagnostic stimulus information and somatomotor systems in embodied, action-oriented representations of emotional meaning[63–67]. Together, these findings are consistent with the possibility that individuals high versus low in stress vulnerability differ less in core affective feelings elicited by non-personalized laboratory stimuli than in how they construe and prepare to respond to aversive events—i.e., in the perception- and action-related relevance assigned to negative scenes. Such individual differences in affective conceptualizations might be more generalizable across experimental tasks, while an additional contribution of affective regions could still emerge if stress induction is assured to be more naturalistic and personally relevant."

"Our preregistered machine learning analyses highlight that not all facets of negative affectivity are equally decodable from negative affective scenes and faces. Anticipating that these tasks are less related to approach-oriented volatility-facets (impulsiveness, angry hostility), we preregistered the qualitative expectation of higher decodability for withdrawal-related facets (stress-vulnerability, depression, anxiety, self-consciousness). Still, the multiverse analysis showed only a predictive advantage of stress vulnerability, not the remaining withdrawal-facets, which was also only apparent for the scenes task. This is highly plausible as the psychological definition of stress vulnerability mirrors the scenes task: How individuals respond to negative events. In contrast, depression is more focused on prolonged affective states and anxiety on the anticipation of threat. Therefore, qualitatively different tasks might better target those facets, like ruminative state switching[68] and threat-of-shock[69], respectively. We nevertheless focused on negative scenes and face viewing because they are the most widely used affective fMRI tasks and are often treated as broadly applicable probes of 'general' negative responsivity, consistent with biological theories of negative affectivity[3,12,14,35]. Using these canonical tasks therefore provides a stringent and maximally field-relevant test of the context-generality assumption, which still underlies current large-scale studies[70]. At the same time, our findings do not exclude that other, more process-selective tasks could yield more accurate predictions of negative affectivity facets or even broad neuroticism. Identifying robust facet-selective task contexts remains an open methodological need for the field. Importantly, one possibility is that broad negative affectivity is "the sum" of facet-specific mechanisms that are each most visible in different, facet-selective task contexts. If so, higher-order traits like neuroticism may only yield coherent neural correlates when aggregating across a battery of such tasks, which would also clarify which latent constructs have a consistent neurobiological counterpart."

COMMENT R4.9

- There are numerous minor typos throughout the manuscript and supplement materials which warrant correction during revision (e.g., "tests" vs. "tested" on page 8, line 165; missing closing parentheses). A careful proofreading pass would improve polish and readability.

Response:

Thank you. These oversights were also pointed out by Reviewer 1 and we tried to carefully re-read the present version and double check proofreading with ChatGPT 5 pro (which was only used for proofing).

COMMENT R4.10

- The addition of the NeuroSynth maps decoding approach in the psychometric assessment paragraph is interesting but appears somewhat unexpected. The motivation for including this particular analysis could be more clearly stated, and it would be helpful if the authors discussed how this approach complements or contrasts with the other analytical strategies employed.

Response:

We added the first and the last sentence to the following paragraph to give more context to the Neurosynth analyses:

“To facilitate the psychological interpretation of the observed neural activation pattern, we conducted automated meta-analytic decoding using Neurosynth[57], which quantifies the association between whole-brain activation maps and large-scale term-based meta-analytic patterns derived from the neuroimaging literature (e.g., “pain”, “fear”, “motoric”). Neurosynth decoding against 50 topic maps from 11,406 fMRI studies indicated that the stress vulnerability pattern has the largest similarity with the broad topic “stimulation” (r = .26) [57]. Hence, the neural pattern might reflect a domain-general neural responsiveness to stimulation in people with higher vulnerability to stress, beyond specifically affective processing”

COMMENT R4.11

- Supplementary Figure S1 may benefit from adjustments to line transparency to better illustrate where most trajectories cluster, as the current presentation makes it difficult to see central tendencies in the data.

Response:

The transparency was increased and we agree with the reviewer that this plot is a much better representation of the data.

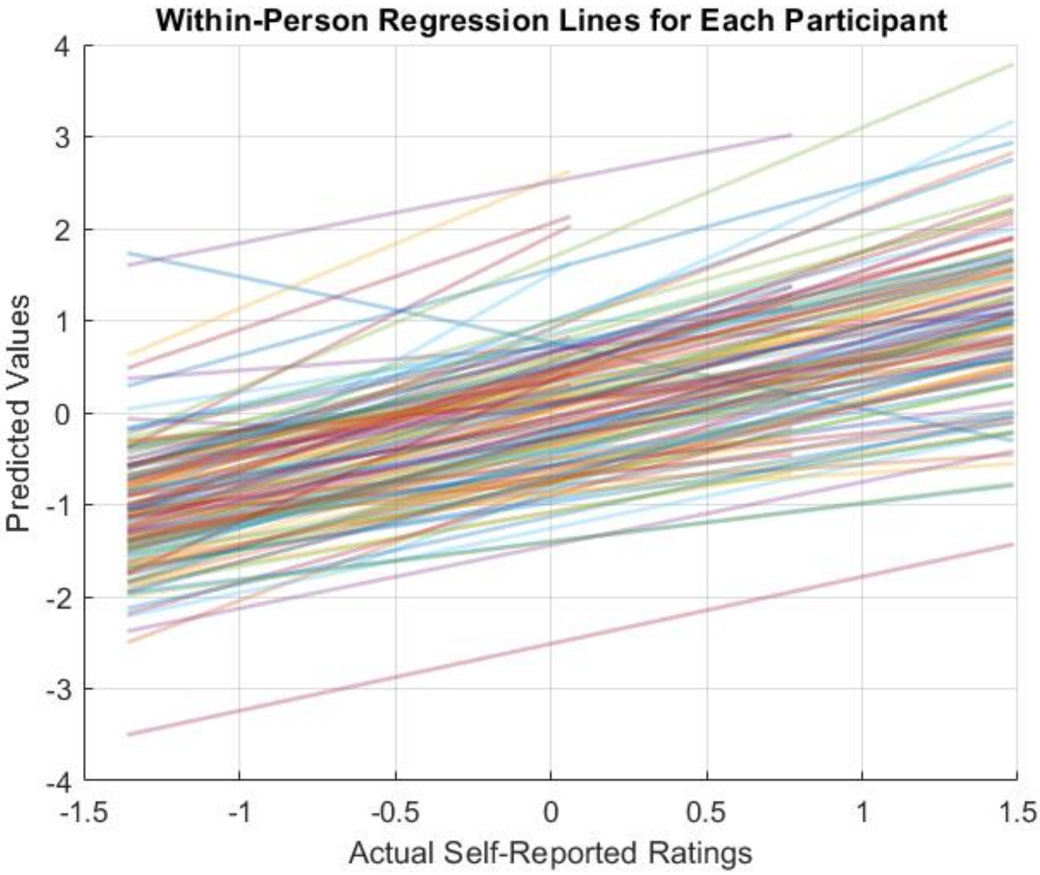

COMMENT R4.12

- In Figure 5b, the neuroticism measures (self-report and other-report) appear to have a smaller number of models compared to other constructs. Maybe I missed it but I’m unsure how this inconsistency is explained.

Response:

This is a very attentive observation! The reason was not provided in the original manuscript. We added this information to the caption:

“Neuroticism scores provided by informants (“Neuroticism: Other”) were only collected in a subset of 165 individuals. Therefore, we only fit these models with all available data, instead of additionally using training data only (Figure 1c; Data choice), leading to a reduced number of models.”

Reviewer #1:

1.The authors have kindly responded to my initial comment that I could not check the preregistration and have now shared it. Thanks for this! However, after inspecting the preregistration document, I realized that there are some significant differences between, for example, the main hypothesis ("A multivariate pattern whole-brain pattern can explain variance in neuroticism") and the hypothesis that the authors state in the manuscript was preregistered ("we preregistered to first predict neuroticism and, if an accuracy threshold is not surpassed, test the predictability of the six lower order facets of neuroticism before evaluating the best model in the hold-out sample"). There is nothing wrong with deviating from the preregistered hypotheses or analyses, but this needs to be clearly acknowledged and reported.

Response: We appreciate the reviewer checking the correctness of the paper regarding the preregistration as well as the other helpful remaining comments!

We tried to disclose all deviations in the previous versions. The specific sentence cited by the reviewer from the manuscript (i.e., focus on sub-facets if condition is not met) is covered by the preregistration under point 3 of section "2.3.2.5 Conditional modelling decisions":

"If correlation accuracy of the final model is not significantly different from zero, the following options will be explored: [...] 3. Outcomes below the factor level of neuroticism will be used as outcomes: a. Neuroticism aspects "withdrawal" and "volatility", computed from the factor scores in. b. If aspects show no significant accuracy, facet scores will be predicted, scored based on the test manual"

In our understanding, the manuscript summarizes this conditional decision structure. As noted in previous manuscript versions, the intermediate aspect level was not pursued further because it would have substantially expanded the model space while the relevant variance was already represented in the more fine-grained facet measures. We acknowledge that also including "conditional hypotheses" in the preregistration would have made this clearer.

2. I still think that several parts of the manuscript could be improved in terms of clarity and readability. To cite only two examples from the abstract: "Evidence including Bayes factors indicated that neuroticism is not associated with any region-, network-, or machine learning-based measure, including amygdala responses. In contrast, a novel brain-wide response pattern robustly predicted the facet stress vulnerability, replicated in an independent dataset". A "brain-wide response pattern" is often derived using machine learning, and therefore the wording sounds contradictory. "...our findings demonstrate the highly selective decodability of 43 emotional dispositions from brain responses". I doubt that most readers not familiar with this literature will understand the meaning of "decodability".

Response: We thank the reviewer for pointing out these specific sections. For the first suggestion, we now phrase it as:

“Evidence including Bayes factors indicated that neuroticism is not associated with any region, network, affective signature, or machine learning pattern, including the amygdala. Still, a brain-wide machine learning pattern robustly predicted the neuroticism facet stress vulnerability (r=.21), replicated in an independent dataset (r=.19). “

Therein, we tried to make it clearer that machine learning was used for both neuroticism and stress vulnerability. For the other suggestion, we changed “decodability” to “predictability”.

3. A number of other expressions and sentences in the manuscript would probably benefit from some revision: “evidence from daily life”, “withdrawal-related facets of neuroticism”, “trait realizations”, “related to approach-oriented volatility-facets”, etc.

Response:

-We changed “evidence from daily life” to “evidence from momentary assessments in daily life”

-For “withdrawal-related facets of neuroticism”, we added “*sub*-facets” to make the hierarchy clearer. In the full manuscript version, it is explicitly explained what these sub-facets are, hopefully further adding clarity: “[...] including depression, anxiety, and stress vulnerability”

-We exchanged “trait *realizations*” for “trait *levels*, from very low to very high, [...]”

-We exchanged “volatility-facets” with “more approach-oriented sub-facets of neuroticism (impulsiveness, angry hostility)”

MINOR/OTHER
“Neurosynth decoding against 50 topic maps from 11,406 fMRI studies indicated that the stress vulnerability pattern has the largest similarity with the broad topic “stimulation” (r = .26). Hence, the neural pattern might reflect a domain-general neural responsiveness to stimulation in people with 245 higher vulnerability to stress, beyond specifically affective processing”
What do the authors mean by “a domain-general neural responsiveness to stimulation”? Use “stress vulnerability” or “vulnerability to stress”; do not use “vulnerability” alone (e.g., in Figure 3).

Response:

-We replaced the second sentence of the following part: “Neurosynth decoding against 50 topic maps from 11,406 fMRI studies indicated that the stress vulnerability pattern has the largest similarity with the broad topic “stimulation” (r = .26). Hence, the neural pattern might reflect a broad, non-specific response profile to general external stimulation that is not limited to affective or visual processing.”

- We checked all instances of “vulnerability” throughout the manuscript and standardized them to “stress vulnerability”
